# Cardiovascular risk factors, cardiovascular disease, and COVID-19: an umbrella review of systematic reviews

**DOI:** 10.1093/ehjqcco/qcab029

**Published:** 2021-06-09

**Authors:** Stephanie L Harrison, Benjamin J R Buckley, José Miguel Rivera-Caravaca, Juqian Zhang, Gregory Y H Lip

**Affiliations:** 1 Liverpool Centre for Cardiovascular Science, University of Liverpool & Liverpool Heart and Chest Hospital, William Henry Duncan Building, 6 West Derby Street, Liverpool L7 8TX, UK; 2 Department of Cardiovascular and Metabolic Medicine, Institute of Life Course and Medical Sciences, University of Liverpool, 6 West Derby Street, Liverpool, L7 8TX, UK; 3 Department of Cardiology, Hospital Clínico Universitario Virgen de la Arrixaca, University of Murcia, Instituto Murciano de Investigación Biosanitaria (IMIB-Arrixaca), CIBERCV, Murcia, Spain; 4 Aalborg Thrombosis Research Unit, Department of Clinical Medicine, Aalborg University, Aalborg, Denmark

**Keywords:** COVID-19 Cardiovascular disease, Cardiovascular risk, Umbrella review

## Abstract

**Aims:**

To consolidate evidence to determine (i) the association between cardiovascular risk factors and health outcomes with coronavirus 2019 (COVID-19); and (ii) the impact of COVID-19 on cardiovascular health.

**Methods and results:**

An umbrella review of systematic reviews was conducted. Fourteen medical databases and pre-print servers were searched from 1 January 2020 to 5 November 2020. The review focused on reviews rated as moderate or high-quality using the AMSTAR 2 tool. Eighty-four reviews were identified; 31 reviews were assessed as moderate quality and one was high-quality. The following risk factors were associated with higher mortality and severe COVID-19: renal disease [odds ratio (OR) (95% confidence interval) for mortality 3.07 (2.43–3.88)], diabetes mellitus [OR 2.09 (1.80–2.42)], hypertension [OR 2.50 (2.02–3.11)], smoking history [risk ratio (RR) 1.26 (1.20–1.32)], cerebrovascular disease [RR 2.75 (1.54–4.89)], and cardiovascular disease [OR 2.65 (1.86–3.78)]. Liver disease was associated with higher odds of mortality [OR 2.81 (1.31–6.01)], but not severe COVID-19. Current smoking was associated with a higher risk of severe COVID-19 [RR 1.80 (1.14–2.85)], but not mortality. Obesity associated with higher odds of mortality [OR 2.18 (1.10–4.34)], but there was an absence of evidence for severe COVID-19. In patients hospitalized with COVID-19, the following incident cardiovascular complications were identified: acute heart failure (2%), myocardial infarction (4%), deep vein thrombosis (7%), myocardial injury (10%), angina (10%), arrhythmias (18%), pulmonary embolism (19%), and venous thromboembolism (25%).

**Conclusion:**

Many of the risk factors identified as associated with adverse outcomes with COVID-19 are potentially modifiable. Primary and secondary prevention strategies that target cardiovascular risk factors may improve outcomes for people following COVID-19.

## Introduction

The coronavirus 2019 (COVID-19) pandemic has caused global health, social, and economic system challenges. As of 14 February 2021, the global cumulative cases of COVID-19 surpassed 108 million; with over 2.3 million related deaths since the start of the pandemic in December 2019.^1^ Early in the COVID-19 pandemic, evidence emerged suggesting that adults with cardiovascular disease (CVD) may be at a higher risk of in-hospital mortality with COVID-19.^2^ In addition, patients hospitalized with COVID-19 demonstrated a propensity to thrombosis.^3^

A rapidly emerging evidence base of COVID-19 research has suggested the following may all increase risk of severe COVID-19 or mortality with COVID-19: higher age, male sex, black or African American or other ethnic minority backgrounds, and underlying health conditions, including CVD and cardiovascular risk factors (e.g. hypertension, diabetes mellitus, and chronic kidney disease).^4–12^ In the UK, a prospective observational cohort study of 20 133 patients hospitalized with COVID-19 suggested that the risk of mortality was higher among patients with cardiac, pulmonary, kidney and liver diseases, as well as cancer, dementia, and obesity (hazard ratios from 1.16 to 1.51).^13^

More severe cases of COVID-19 have been associated with new-onset cardiovascular conditions. Although, the hallmark of COVID-19 is respiratory involvement, ranging from mild upper respiratory symptoms to acute respiratory distress syndrome,^14^ severe COVID-19 has been implicated in multi-organ involvement, with several observational case series showing a significant proportion of cardiac involvement among hospitalized patients.^9^^,^^15^^,^^16^ COVID-19 appears to associate with a wide spectrum of cardiovascular sequelae, including acute-onset heart failure, arrhythmias, acute coronary syndrome, myocarditis, and cardiac arrest. Moreover, the acute cardiac injury seems to be significantly correlated with increased in-hospital mortality in COVID-19 patients.^9^

Despite our current understanding of CVD and outcomes with COVID-19, the rapid expanse of research during the pandemic has resulted in substantial variability of risk estimates and duplication of patient data. Indeed, this is particularly problematic when focusing on systematic reviews and meta-analyses, arguably the gold standard of scientific evidence, which have included overlapping patient populations and represent a variety of evidence quality.

Commissioned by Public Health England, the aim of this umbrella review of systematic reviews was to consolidate evidence which addressed the following two research questions: (i) What is the association between cardiovascular risk factors or CVD and health outcomes, hospitalization, ventilation, and mortality caused by COVID-19? and (ii) What is the impact of COVID-19 on cardiovascular health?

## Methods

This umbrella review was conducted using the Preferred Reporting Items for Systematic Reviews and Meta-Analyses (PRISMA) guidelines.^17^ Although there was no published protocol, the research questions, search strategy, and inclusion/exclusion criteria were independently developed by Public Health England prior to the commencement of the review.

### Objectives

To determine (i) the association between cardiovascular risk factors or CVD and health outcomes with COVID-19; and (ii) the impact of COVID-19 on cardiovascular health.

### Population, exposure, comparator, and outcomes

The population was people with COVID-19. Reviews which were focused on children (aged <18 years) were not eligible for inclusion. For the first objective, the exposures were cardiovascular risk factors, CVD, or cerebrovascular disease. Cardiovascular risk factors pre-defined as eligible for inclusion were smoking, hypertension, obesity, sedentary behaviour/physical inactivity, alcohol use, diet, cholesterol, familial hypercholesterolaemia, hyperlipoproteinaemia type II, hyperglycaemia, prediabetic state, diabetes, atrial fibrillation, renal insufficiency, kidney diseases, liver diseases, fibrosis, and dementia. The comparator group included individuals with COVID-19 without CVD or the risk factor of interest. The outcomes were any health outcomes with COVID-19 including hospitalization, ventilation, and mortality, or composite measures of these. No exclusions were placed on methods used to diagnose COVID-19, CVD, or cardiovascular risk factors. For the second objective, the exposure was COVID-19 and a comparator group was not needed. The outcomes were any incident cardiovascular or cerebrovascular events following a diagnosis of COVID-19.

### Study design

Systematic reviews or meta-analyses were eligible for inclusion. In accordance with the Database of Abstracts of Reviews of Effects (DARE) criteria, to be included, the reviews needed to have detailed the inclusion and exclusion criteria, conducted an adequate search, assessed the quality of included studies, synthesized the results of the included studies and provided sufficient details of the characteristics of the included studies.^18^ Pre-prints, grey literature, or peer-reviewed publications were eligible for inclusion. Where a pre-print and a peer-reviewed publication of the same systematic review were found, only the peer-reviewed publication was included. Only reviews published in the English language were eligible for inclusion.

### Search strategy

The search was conducted in early November 2020, and the following electronic databases were searched from 1 January 2020 to 5 November 2020: Cochrane Library, Ovid Medline, Ovid Emcare, Embase, Epistemonikos COVID-19, EPPI Living Map, Evidence Aid, Global Health, LENUS, medRxiv, Norwegian Institute of Public Health, PROSPERO, PubMed, and the World Health Organisation. Exploded Medical subject headings (MesH) terms were combined with appropriate free-text terms for CVD, cardiovascular risk factors, and COVID-19. These were mapped across different databases. The Medline systematic review search filter was applied to the search to limit the number of results to this type of review. The search strategy conducted in Medline is shown in Supplementary material online, *Table S1*.

### Study selection

The results from the different electronic databases were exported into EndNote X9 and duplicates were removed. Two reviewers (S.L.H. and B.J.R.B.) completed title and abstract screening independently and in duplicate. Of the potentially included reviews, full-texts were retrieved and also independently screened in duplicate by the same two reviewers to identify reviews for inclusion. Disagreements were resolved through discussion to reach a consensus.

### Data extraction

A data extraction form was pre-defined in Microsoft Excel with the following information: first author, review search dates, number of included studies, countries of included studies, study designs of included studies, number of patients, population inclusion criteria, exposures examined, outcomes examined, whether a meta-analysis was performed (yes/no), methods if a meta-analysis was performed (e.g. random-effects or fixed-effects model), results for each exposure and outcome of interest (and number of studies and patients for each analysis if different from the total study sample), quality assessment results, conclusions, and reported limitations. The principal summary measures were odds ratios, risk ratios, or hazard ratios. Two reviewers (S.L.H. and B.J.R.B.) independently completed the data extraction in duplicate for 10 of the reviews (12%) and achieved good agreement (≥80%). Data extraction for the remaining included reviews was completed by one reviewer (S.L.H. or B.J.R.B.). This approach is in line with the AMSTAR 2 checklist.^19^

### Quality assessment

Two reviewers (J.M.R.-C. and J.Z.) independently critically assessed the quality of 10 included reviews (12%) using the AMSTAR 2, which is a critical appraisal tool for systematic reviews that include randomized or non-randomized studies of healthcare interventions.^19^ The reviewers discussed any disagreement until optimal agreement was achieved (100%), and the quality assessment of the remaining included reviews was completed by one reviewer (J.M.R.-C. or J.Z.). The AMSTAR 2 includes 16 items, and as the AMSTAR 2 is designed for reviews of interventions, we modified the items which referred to ‘interventions’ to refer to ‘exposures’ in the included reviews. Using the AMSTAR 2 checklist, each included review was given an overall confidence rating of ‘critically low’ (more than one critical flaw with or without non-critical weaknesses), ‘low’ (one critical flaw with or without non-critical weaknesses), ‘moderate’ (more than one non-critical weakness) or ‘high’ (no or one non-critical weakness).

### Overlapping reviews

Reviews overlapped if they examined associations between the same exposure and outcome. It was noted that duplication of primary studies within reviews which examined the same exposure and outcome was extensive; therefore, incorporating results from reviews which examined the same exposure and outcomes was likely to lead to the inclusion of the same primary studies more than once. More recent reviews tended to include larger numbers of patients, greater numbers of cohort studies, and data from a wider variety of countries. Therefore, for each risk factor included in this umbrella review, we have highlighted the findings from the largest moderate- or high-quality systematic review.

### Data synthesis

A narrative synthesis of the included systematic reviews and meta-analyses was conducted. Summary tables describe review characteristics and findings of meta-analyses are presented using forest plots.

## Results

### Screening

**Figure 1:**
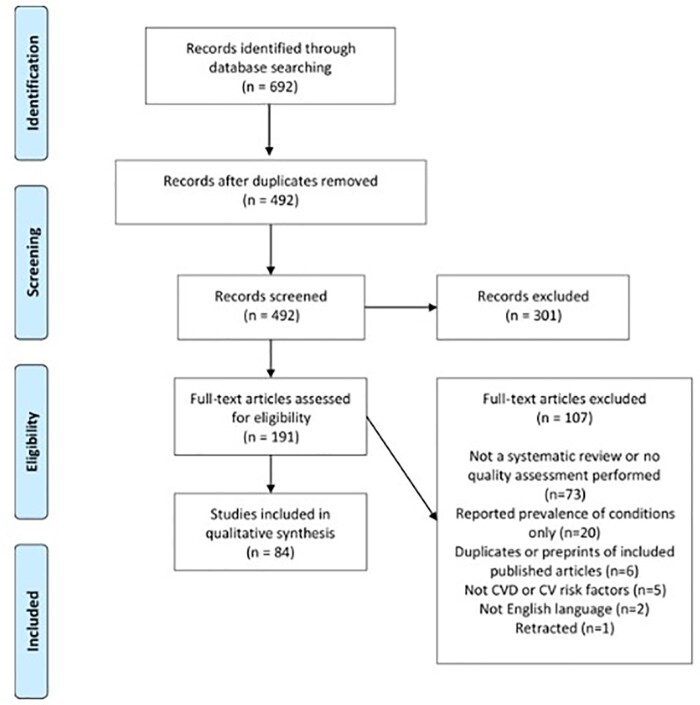
PRISMA flow diagram.

The searches resulted in 692 studies identified and after the removal of duplicates, 492 studies were screened at the title and abstract stage (*Figure 1*). After reviewing the title and abstracts, 301 (61.2%) were removed, and the full-texts were retrieved for 191 studies and subsequently assessed for eligibility. At the full-text screening stage, 107 articles were excluded and the reasons are listed in the PRISMA flow diagram. Attempts were made to contact the authors of one of the included reviews for further information, but no response was received. After the full-text screening, 84 systematic reviews or meta-analyses which addressed the research questions were identified.

### Assessment of the quality of the included reviews

Of the 84 included reviews, according to the AMSTAR 2 rating, 33% (*n* = 28 reviews) were assessed as critically low quality; 29% (*n* = 24) were assessed as low quality, 37% (*n *= 31) were moderate quality,^20–50^ and only one review, which reported associations between smoking and outcomes with COVID-19, was assessed as high-quality.^51^ The full AMSTAR 2 assessments and references to the critically low and low-quality studies are provided in Supplementary material online, Table S2.

The AMSTAR 2 criteria which were often met by the included reviews were: (i) outlining the research questions and inclusion criteria including the elements of participants, intervention, comparator group, and outcomes (AMSTAR 2 criterion #1), (ii) explaining the selection of study designs for inclusion (AMSTAR 2 criterion #3), (iii) performing duplicate screening and duplicate data extraction (AMSTAR 2 criteria #5 and #6), and (iv) declaring potential conflicts of interest and sources of funding (AMSTAR 2 criterion #16). Most of the reviews achieved ‘Partially Yes’ in the following sections: assessing selection bias and confounding factors in the risk of bias assessment (AMSTAR 2 criterion #9), and providing sufficient explanation on the method prior to its conduction (AMSTAR 2 criterion #2), although limited studies reported clear plans to investigate causes of heterogeneity. Although most reviews mentioned publication bias or planned for its assessment (AMSTAR 2 criterion #15), some of the reviews did not perform an assessment of this bias or report this bias either due to small sample size, or no explanation was provided.

The AMSTAR 2 criteria which were not reported in the majority of the reviews were (i) a list of excluded studies and justifications for exclusion (AMSTAR 2 criterion #7) and (ii) reporting sources of funding for studies included in the review (AMSTAR 2 criterion #10). Further, AMSTAR 2 criteria which were often not fulfilled in the included reviews were justification of restrictions of the search, adjustment for confounding factors in meta-analyses, assessment on the impact of risk of bias for individual studies on the results of the meta-analysis, and sufficient discussion and interpretation of the results with the impact of individual risk of bias.

### Characteristics of the moderate and high-quality reviews

The number of studies in the included 32 moderate or high-quality reviews ranged from 3^45^ to 75.^38^ The earliest search date was to 2 March 2020,^26^ and the most recent search date was to 11 August 2020.^39^ Three reviews only included studies from China,^22^^,^^26^^,^^46^ two reviews did not report the country of the studies^24^^,^^36^ and 27 reviews included studies from multiple countries. The reviews included observational studies such as case reports, case series, cross-sectional studies, and retrospective and prospective cohort studies. Supplementary material online, *Table S3* summarizes the characteristics and results of all 84 reviews identified.

### Cardiovascular disease or cardiovascular risk factors and outcomes with COVID-19

**Figure 2:**
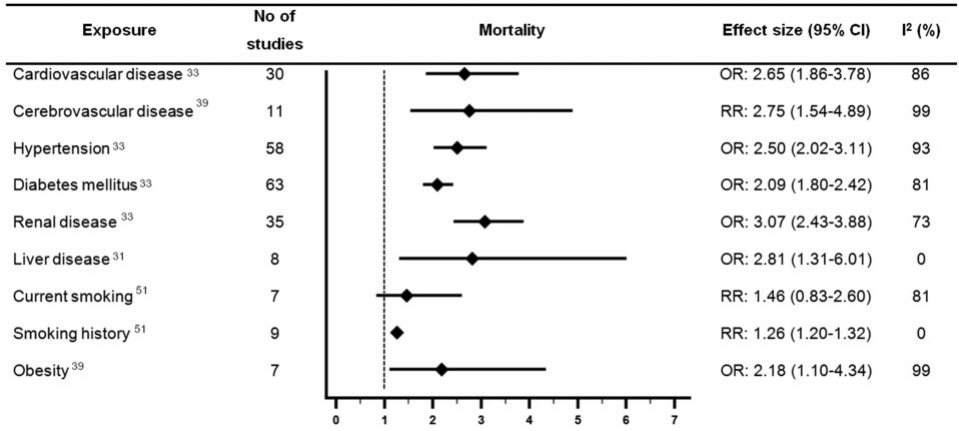
Forest plot showing results of meta-analyses from reviews which investigated associations between cardiovascular disease or cardiovascular risk factors and mortality with COVID-19. Largest moderate- or high-quality review included, according to assessment with the AMSTAR 2 criteria. CI, confidence interval; OR, odds ratio; RR, relative risk.

**Figure 3:**
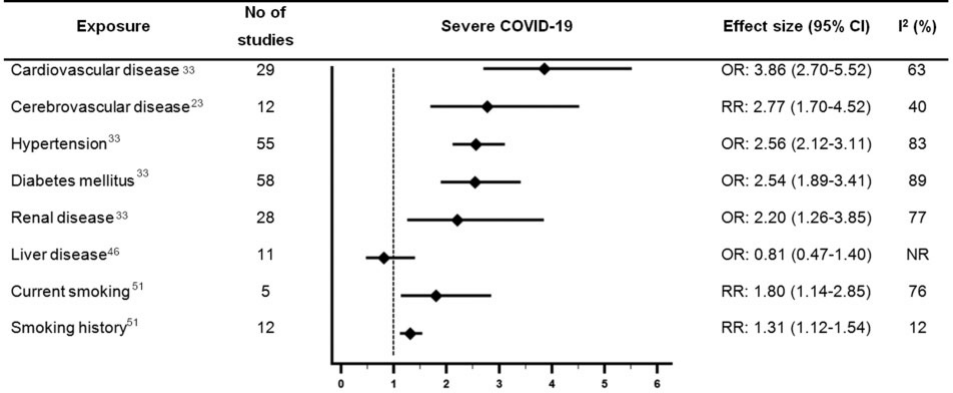
Forest plot showing results of meta-analyses from reviews which investigated associations between cardiovascular disease or cardiovascular risk factors and severe COVID-19. Largest moderate- or high-quality review included, according to assessment with the AMSTAR 2 criteria. CI, confidence interval; NR, not reported; OR, odds ratio; RR, relative risk.

*Figures 2 and 3* provide a summary of the main findings of moderate or high-quality reviews which examined associations between CVD or cardiovascular risk factors and outcomes with COVID-19. *Figures 2 and* *3* focus on the outcomes which were most consistently reported in the included reviews (mortality and severe COVID-19). However, there was variation in how severe COVID-19 was defined (Supplementary material online, *Table S3*).

#### Cardiovascular disease

Ten moderate quality systematic reviews examined associations between CVD and outcomes with COVID-19.^20^^,^^21^^,^^23^^,^^31^^,^^33^^,^^36^^,^^38^^,^^39^^,^^44^^,^^49^ The largest of which (Luo *et al*.,) suggested CVD was associated with 2.65 times higher odds of mortality with COVID-19 [pooled odds ratio (OR) 2.65, 95% confidence interval (CI) 1.86–3.78, *n* = 30 studies, considerable heterogeneity (I^2^ = 86%)].^33^

Five reviews examined the association between CVD and severe COVID-19 and were rated as moderate quality.^23^^,^^24^^,^^33^^,^^35^^,^^48^ However, the definition of severe COVID-19 was inconsistent across the reviews. The largest of which (Luo *et al*.,) suggested CVD was associated with 3.86 times higher odds of severe COVID-19 [pooled OR 3.86 (2.70–5.52), *n *= 29 studies, substantial heterogeneity (*I*^2^ = 63%)].^33^

Three reviews examined associations between coronary heart disease (CHD) and outcomes with COVID-19 and were rated moderate quality.^20^^,^^23^^,^^39^ The largest review included 11 studies and suggested CHD was significantly associated with 3.63 times higher odds of mortality with COVID-19 [pooled OR 3.63 (1.52–8.65), considerable heterogeneity (*I*^2^ = 100%)].^39^ One review which was rated moderate quality reported CHD was associated with 2 times higher odds of severe COVID-19 [pooled OR 2.03 (1.39–2.97), moderate heterogeneity (*I*^2^ = 44%)].^23^

#### Cerebrovascular disease

Nine reviews which were rated as moderate quality examined associations between cerebrovascular disease and outcomes with COVID-19.^21^^,^^23^^,^^25^^,^^31^^,^^38^^,^^39^^,^^44^^,^^46^^,^^48^ The moderate quality review with the largest number of studies reported cerebrovascular disease was associated with a significant 2.75 times higher risk of mortality [pooled relative risk (RR) 2.75 (1.54–4.89), *n* = 11 studies, considerable heterogeneity (*I*^2^ = 99%)].^39^ The review by Fang *et al*.,^23^ was also rated moderate quality and included the largest number of studies examining the association between cerebrovascular disease and severe COVID-19. Fang *et al*.,^23^ reported that cerebrovascular disease was associated with 2.77 times higher risk of severe COVID-19 [pooled RR 2.77 (1.70–4.52), *n* = 12 studies, moderate heterogeneity (*I*^2^ = 40%)].

Two moderate quality reviews examined the association between cerebrovascular disease and intensive care unit (ICU) admission with COVID-19. The largest of these reviews did not find a significant association between cerebrovascular disease and risk of ICU admission with COVID-19 [pooled RR 1.9 (0.9–4.0), *n *= 4 studies, considerable heterogeneity (*I*^2^ = 92%)].^38^

It was unclear in the majority of the reviews if the stroke occurred prior to or following a COVID-19 diagnosis.

#### Hypertension

Of the reviews which examined hypertension, 15 were rated as moderate quality.^20^^,^^21^^,^^23^^,^^24^^,^^30–33^^,^^35^^,^^36^^,^^38^^,^^39^^,^^44^^,^^46^^,^^48^ Of the moderate quality reviews, Luo *et al*.,^33^ included the largest number of studies and suggested hypertension was associated with 2.5 times higher odds of mortality [pooled OR 2.50, (2.02–3.11), *n* = 58 studies, considerable heterogeneity (*I*^2^ = 93%)]. Luo *et al*. also reported a significant association between hypertension and higher odds of severe COVID-19 [pooled OR 2.56 (2.12–3.11), *n* = 55 studies, considerable heterogeneity (*I*^2^ = 83%)]. One moderate quality review reported a significant association between hypertension and higher odds of a composite adverse outcome of mortality, mechanical ventilation, or severe COVID-19 [pooled OR 3.15 (2.26–4.41), *n* = 38 studies, moderate heterogeneity (*I*^2^ = 40%)].^24^ Two moderate quality reviews examined the association between hypertension and risk of ICU admission,^23^^,^^38^ with the largest, more recent review reporting a pooled RR of 1.4 (1.1–1.7), *n* = 9 studies, and substantial heterogeneity (*I*^2^ = 53%).^38^ Associations between antihypertensive medication use and outcomes with COVID-19 were not examined in this umbrella review.

#### Diabetes mellitus

Of the reviews which examined diabetes mellitus, 18 were rated as moderate quality.^20^^,^^21^^,^^23^^,^^24^^,^^30–36^^,^^38–40^^,^^44^^,^^46–48^ Luo *et al*., conducted the largest moderate quality review and reported significant associations between diabetes mellitus and higher odds of mortality [pooled OR 2.09 (1.80–2.42), *n* = 63 studies, considerable heterogeneity (*I*^2^ = 81%)], and severe COVID-19 [pooled OR 2.54 (1.89–3.41), *n* = 58 studies, considerable heterogeneity (*I*^2^ = 89%)].^33^ One moderate quality review reported a significant association between diabetes mellitus and 2.34 times higher odds of a composite adverse outcome of mortality, mechanical ventilation or severe COVID-19 [pooled OR 2.34 (1.64–3.33), *n* = 34 studies, substantial heterogeneity (*I*^2^ = 80%)].^24^ Two moderate quality reviews examined the association between diabetes and risk of ICU admission,^23^^,^^38^ and the largest, more recent review reported a pooled RR of 1.9 (1.4–2.6), *n* = 12 studies, and considerable heterogeneity (*I*^2^ = 90%)].^38^ The association between diabetes and mortality with COVID-19 was stratified by age group in one moderate quality review of nine studies, and the association only remained statistically significant for patients aged <70 years [pooled OR 2.05 (1.44–2.94), moderate heterogeneity (*I*^2^ = 32%)].^47^

#### Renal disease

All reviews compared renal/kidney disease/disorder or chronic kidney disease to no renal disease/disorder. No reviews examined the impact of different stages of renal disease on outcomes with COVID-19. Eight reviews rated as moderate quality examined associations between renal disease and outcomes with COVID-19.^20^^,^^23^^,^^31^^,^^33^^,^^38^^,^^39^^,^^44^^,^^48^ The largest moderate quality review reported a significant association between renal disease and higher odds of mortality [pooled OR 3.07 (2.43–3.88), *n* = 35 studies, substantial heterogeneity (*I*^2^ = 73%)],^33^ and severe COVID-19 [pooled OR 2.20 (1.26–3.85), *n *= 28 studies, considerable heterogeneity (*I*^2^ = 77%)).^33^

#### Liver disease

Six reviews rated as moderate quality examined associations between liver disease and outcomes with COVID-19. Some of the reviews referred to ‘chronic liver disease’ whilst others only specified ‘liver disease’, and no distinctions were made for the severity of liver disease. Four moderate quality reviews examined the association between liver disease and mortality with COVID-19.^31^^,^^38^^,^^39^^,^^44^ Islam *et al*.^31^ was the largest review and reported a significant association between liver disease and 2.81 times higher odds of mortality [pooled OR 2.81 (1.31–6.01), *n* = 8 studies, no heterogeneity (*I*^2^ = 0%)]. The largest moderate quality review which examined the association between liver disease and severe COVID-19 did not find a significant association [pooled OR 0.81 (0.47–1.40), *n* = 11 studies, *I*^2^ not reported], but this review was relatively older (searches until April 2020).^46^

#### Obesity

Three reviews rated as moderate quality examined associations between obesity or body mass index (BMI) and outcomes with COVID-19.^39^^,^^45^^,^^46^ The largest moderate quality review reported a statistically significant association between obesity and mortality [pooled OR 2.18, (1.10–4.34), *n* = 7 studies considerable heterogeneity (*I*^2^ = 99%)].^39^ One further moderate quality review also suggested obesity was associated with increased risk of in-hospital critical care with COVID-19, but a meta-analysis was not performed.^45^ One moderate quality review including only four studies of 221 patients reported no statistically significant association between BMI and severe COVID-19.^46^

#### Smoking

One of the reviews which examined smoking and outcomes with COVID-19 was rated high-quality,^51^ and six reviews were rated moderate quality.^21^^,^^28^^,^^35^^,^^39^^,^^41^^,^^46^ There were differences in the comparisons made in the reviews which examined smoking (e.g. current/former smoker vs. never smoker and current smoking vs. not current smoking). The high-quality review by Reddy *et al*.,^51^ reported a statistically significant association between current smoking and 1.80 times higher risk of severe COVID-19 [pooled RR 1.80 (1.14–2.85), *n* = 5 studies, considerable heterogeneity (*I*^2^ = 76%)], but no significant association between current smoking and disease progression, ICU admission, mechanical ventilation, or mortality. One moderate quality review which examined smoking had a more recent search date than the high-quality review and also did not find a significant association between current smoking and mortality with COVID-19.^39^

In the same high-quality review, smoking history vs. never smoking was associated with severe COVID-19 [pooled RR 1.31 (1.12–1.54), *n* = 12 studies, low heterogeneity (*I*^2^ = 12%)], disease progression [pooled RR 2.18 (1.06–4.49), *n* = 5 studies, substantial heterogeneity (*I*^2^ = 69%)], mechanical ventilation [pooled RR 1.20 (1.01–1.42), *n* = 4 studies, no heterogeneity (*I*^2^ = 0%)] and mortality [pooled RR 1.26 (1.20–1.32), *n* = 9 studies, no heterogeneity (*I*^2^ = 0%)], but not ICU admission.^51^

#### Alcohol

One moderate quality review was identified which examined associations between alcohol consumption and severe COVID-19.^46^ The review only identified one study which included 30 patients with COVID-19 and did not find a statistically significant association between alcohol use and severe COVID-19 [OR 1.86 (0.40–8.69)].

#### Multiple cardiovascular risk factors

One moderate quality review examining 21 studies with >77 000 patients reported a significant association between increasing numbers of cardiovascular co-morbidities or cardiovascular risk factors and higher mortality was significantly associated with COVID-19 case fatality rate (regression coefficient 0.001, 95% CI 0.003–0.005, *P* < 0.001).^42^

### The impact of COVID-19 on cardiovascular health

All of the reviews which examined the impact of COVID-19 on cardiovascular health were completed in the acute phase, and no reviews were found which examined the impact of COVID-19 on longer-term cardiovascular outcomes.

#### Acute cardiac injury

Five reviews rated as moderate quality reported the pooled incidence of acute cardiac injury in patients with COVID-19.^27^^,^^30^^,^^36^^,^^37^^,^^42^ Of these reviews, only two further defined acute cardiac injury.^27^^,^^37^ One of the reviews defined acute cardiac injury as ‘serum levels of troponin or CK-MB above the 99th percentile upper reference limit, regardless of new abnormalities in electrocardiography and echocardiography’,^27^ and one review defined acute cardiac injury as ‘troponin levels >28 pg/mL’.^37^ Amongst these reviews the incidence of acute cardiac injury ranged from 6%^30^ to 25%.^36^ The largest moderate quality review included over 77 000 participants and suggested the incidence of acute myocardial injury was 10.3%.^42^

Four reviews rated as moderate quality examined the association between acute cardiac injury and outcomes with COVID-19.^24^^,^^33^^,^^36^^,^^48^ The largest, most recent moderate quality review reported an association between acute cardiac injury and 17 times higher odds of mortality with COVID-19 [pooled OR 16.97 (7.87–36.57), *n* = 14 studies, considerable heterogeneity (*I*^2^ = 89%)].^33^

One moderate quality review reported a significant association between acute cardiac injury and a composite adverse outcome of mortality, mechanical ventilation, or severe COVID-19 [pooled OR 10.58 (5.00–22.40), *n* = 12 studies, substantial heterogeneity (*I*^2^ = 59%)].^24^ Two moderate quality reviews examined the association between acute cardiac injury and severe COVID-19.^33^^,^^48^ The largest, more recent moderate quality review reported a significant association between acute cardiac injury and severe COVID-19 [pooled OR 6.57 (3.70–11.65), *n* = 11 studies, considerable heterogeneity (*I*^2^ = 75%)].^33^

#### Incident venous thromboembolism, pulmonary embolism, and deep vein thrombosis

One moderate quality meta-analysis of 17 studies estimated the incidence of venous thromboembolism (VTE), pulmonary embolism, and deep vein thrombosis as 25% (95% CI: 19%-31%), 19% (13%-25%) and 7% (4%–10%), respectively for patients hospitalized with COVID-19. All of these estimates were shown to have considerable heterogeneity. A higher incidence of VTE was observed in severe compared to non-severe patients (pooled RR 4.76 (2.66–8.50), moderate heterogeneity (*I*^2^ = 47%)).^50^

#### Incident arrhythmia, cardiac failure, and cerebrovascular disease

Two reviews rated moderate quality examined the incidence of arrhythmia developed during hospitalization with COVID-19.^29^^,^^42^ The larger of these reported incidence of arrhythmias as 18.4% (95% CI 7.8–32.3%).^42^ One moderate quality review of four studies (245 patients) estimated the incidence of acute cardiac failure with COVID-19 as 6.5% (2.2%–12.2%), considerable heterogeneity (*I*^2^ = 78%).^26^ The incidence of acute cerebrovascular disease was not examined in any reviews rated as moderate quality.

#### Incident composite cardiovascular complications

Two moderate quality reviews examined the incidence of any cardiovascular complication with COVID-19.^42^^,^^43^ The most recent moderate quality review reported a pooled incidence of 14.1% (10.3–20.2%) for any cardiovascular complication developed in-hospital with COVID-19.^42^ This review also reported a statistically significant association between cardiovascular complications and COVID-19 case fatality rate (regression coefficient 0.001, 95% CI 0.000–0.003, *P *= 0.038).^42^

## Discussion

Evidence from this umbrella review suggests CVD, hypertension, diabetes mellitus, renal disease, and smoking history associate with a higher likelihood of severe COVID-19 and mortality with COVID-19. Current smoking associated with significantly higher risk of severe COVID-19, but not mortality. Liver disease was associated with significantly higher odds of mortality, but not severe COVID-19. Obesity associated with higher odds of mortality with COVID-19. Although cerebrovascular disease was associated with a higher likelihood of adverse outcomes with COVID-19, it was often unclear if the stroke occurred prior to or following infection. There was insufficient evidence to make conclusions regarding alcohol consumption and outcomes with COVID-19. Furthermore, although an extensive search was conducted, no moderate quality reviews were identified which examined cholesterol levels, arrhythmias, diet, physical activity, or dementia and outcomes with COVID-19. No identified reviews examined the impact of cardiovascular health on long-COVID.

Acute heart failure following COVID-19 was 2%, the incidence of myocardial infarction was 4%, myocardial injury was 10%, angina was 10%, arrhythmias was 18%, and incidence of venous thromboembolism, pulmonary embolism, and deep vein thrombosis was 25%, 19%, and 7%, respectively. No identified reviews examined the impact of COVID-19 on long-term cardiovascular health.

Early in the COVID-19 pandemic, concerns were raised about the potential for COVID-19 to cause new-onset cardiovascular complications or exacerbate existing CVD because of prior knowledge from influenza epidemics and outbreaks of other respiratory viruses.^52^ Influenza epidemics have been associated with a rise in cardiovascular mortality.^53^ Emerging evidence has suggested the COVID-19 pandemic may also have resulted in excess deaths in people with CVD, but this may be due to direct effects of infection and indirect effects due to changes in availability of healthcare and behaviour changes.^54^

The higher proportion of adverse outcomes with COVID-19 for patients with CVD or cardiovascular risk factors may also be due to underlying changes these risk factors have caused on inflammatory pathways, immune function, and/or lung function. First, Influenza and other upper respiratory infections may result in cardiovascular complications by causing a pro-inflammatory state.^53^^,^^55^ Similarly, dysregulation of coagulation and a hyperinflammatory response has been reported in some patients with COVID-19, which may cause a ‘hypercoagulable state’ and a propensity for thrombosis and thrombosis-related complications.^3^^,^^56^ Second, cardiovascular risk factors such as diabetes mellitus may lead to immune function dysregulation, which may in turn increase susceptibility and predispose these patients to severe presentation of COVID-19.^57^ Third, some cardiovascular risk factors including smoking and obesity may impair pulmonary function resulting in a higher risk of severe outcomes with COVID-19. Both current and former smokers have accelerated lung function decline compared to never-smokers.^58^ Additionally, obesity may also impair lung function by pushing the diaphragm upward and reducing lung volume.^59^

Identifying cardiovascular risk factors for worsened COVID-19 prognosis is important to identify high-risk patient groups and targeting of intervention strategies. Many of the risk factors identified as significantly associated with adverse outcomes with COVID-19 are potentially modifiable. Therefore, primary and secondary prevention strategies which target these cardiovascular risk factors and conditions may improve outcomes for people following COVID-19.

Further research should focus on the impact of multiple cardiovascular risk factors and other multi-morbidity associations with COVID-19, as cardiovascular risk factors rarely occur in isolation. Although evidence will be observational, further research should include appropriate adjustment for confounding factors including age, socioeconomic status, and ethnicity, which are established risk factors for COVID-19 severity.

All included reviews which examined the impact of COVID-19 on cardiovascular health observed in-hospital cardiovascular outcomes only, and the impact of COVID-19 on long-term cardiovascular health was not investigated. Future research should determine longer-term outcomes for people with COVID-19, including whether COVID-19 impacts incident cardiovascular and cerebrovascular outcomes post-discharge, and whether CVD associates with a higher risk of long-COVID.

### Strengths and limitations

This umbrella review included a systematic search strategy to examine a wide-range of cardiovascular risk factors and cardiovascular conditions in relation to outcomes with COVID-19, and the impact of COVID-19 on cardiovascular health. Cardiovascular biomarkers were not included and the impact of treatments for COVID-19 on the observed associations was not examined as this was beyond the scope of the current review. Only reviews available in the English language were included. The quality of the included reviews varied, 52 critically low- and low-quality reviews according to the AMSTAR 2 checklist were included and there was duplication of primary studies within the reviews. However, we have focused on the results of reviews which were rated as moderate and high-quality. Due to the nature of the research questions, only observational evidence was available to address the questions, which typically provides low certainty evidence and cannot infer causality. Confounding factors such as age, sex, and ethnicity may impact the results of reviews, but it was not clear in many of the reviews if the studies included in meta-analyses adjusted for these factors. Furthermore, high levels of heterogeneity were often reported in meta-analyses, which was typically not further investigated. Within the included reviews, there were inconsistencies in definitions used for severe COVID-19. Pre-prints were included because of the rapidly emerging evidence base, but the results reported in these articles may be subject to change following peer-review. However, only one of the reviews included in the forest plots was a pre-print.^31^

## Conclusions

In conclusion, the evidence reported in this umbrella review suggests that CVD and certain cardiovascular risk factors including hypertension, diabetes mellitus, renal disease, liver disease, cerebrovascular disease, obesity, smoking history and current smoking associated with a higher likelihood of severe COVID-19 and/or mortality with COVID-19. Incident cardiovascular complications following hospitalization with COVID-19 may be up to 25% depending on the complication, but further evidence is needed to determine the impact of COVID-19 on long-term cardiovascular health outcomes. Many of the cardiovascular risk factors associated with COVID-19 are modifiable. Clinicians and policy makers should consider that primary and secondary prevention strategies which improve cardiovascular health may also improve outcomes for people following COVID-19.

## Supplementary material

Supplementary material is available at *European Heart Journal – Quality of Care and Clinical Outcomes* online.

## Data availability

No new data were generated or analysed in support of this research.

## Supplementary Material

qcab029_Supplementary_Data

## References

[1] WHO. *COVID-19 Weekly Epidemiological Update*. 2021. Available at: https://www.who.int/publications/m/item/weekly-epidemiological-update---16-february-2021 (last accessed 7 May 2021).

[2] Mehra MR , Desai SS, Kuy S, Henry TD, Patel AN. Cardiovascular disease, drug therapy, and mortality in Covid-19. N Engl J Med 2020;382:e102. 32356626 10.1056/NEJMoa2007621 PMC7206931

[3] Bikdeli B , Madhavan MV, Jimenez D, Chuich T, Dreyfus I, Driggin E et al COVID-19 and thrombotic or thromboembolic disease: implications for prevention, antithrombotic therapy, and follow-up. J Am Coll Cardiol 2020;75:2950–2973. 32311448 10.1016/j.jacc.2020.04.031 PMC7164881

[4] Yang X , Yu Y, Xu J, Shu H, Xia J, Liu H et al Clinical course and outcomes of critically ill patients with SARS-CoV-2 pneumonia in Wuhan, China: a single-centered, retrospective, observational study. Lancet Respir Med 2020;8:475–481. 32105632 10.1016/S2213-2600(20)30079-5 PMC7102538

[5] Yancy CW. COVID-19 and African Americans. JAMA 2020;323:1891. 32293639 10.1001/jama.2020.6548

[6] Rimmer A. Covid-19: two thirds of healthcare workers who have died were from ethnic minorities. BMJ 2020;369:m1621. 32327412 10.1136/bmj.m1621

[7] Richardson S , Hirsch JS, Narasimhan M, Crawford JM, McGinn T, Davidson KW, the Northwell C-RC et al Presenting characteristics, comorbidities, and outcomes among 5700 patients hospitalized with COVID-19 in the New York City Area. JAMA 2020;323:2052. 32320003 10.1001/jama.2020.6775 PMC7177629

[8] Du R-H , Liang L-R, Yang C-Q, Wang W, Cao T-Z, Li M et al Predictors of Mortality for patients with COVID-19 pneumonia caused by SARS-CoV-2: a prospective cohort study. Eur Respir J 2020;55:2000524. 32269088 10.1183/13993003.00524-2020 PMC7144257

[9] Shi S , Qin M, Shen B, Cai Y, Liu T, Yang F et al Association of cardiac injury with mortality in hospitalized patients with COVID-19 in Wuhan, China. JAMA Cardiol 2020;5:802. 32211816 10.1001/jamacardio.2020.0950 PMC7097841

[10] Henry BM , Lippi G. Chronic kidney disease is associated with severe coronavirus disease 2019 (COVID-19) infection. Int Urol Nephrol 2020;52:1193–1194. 32222883 10.1007/s11255-020-02451-9 PMC7103107

[11] Clark A , Jit M, Warren-Gash C, Guthrie B, Wang HHX, Mercer SW et al Global, regional, and national estimates of the population at increased risk of severe COVID-19 due to underlying health conditions in 2020: a modelling study. The Lancet Global Health 2020;8:e1003–e1017. 32553130 10.1016/S2214-109X(20)30264-3 PMC7295519

[12] Williamson EJ , Walker AJ, Bhaskaran K, Bacon S, Bates C, Morton CE et al Factors associated with COVID-19-related death using OpenSAFELY. Nature 2020;584:430–436. 32640463 10.1038/s41586-020-2521-4 PMC7611074

[13] Docherty AB , Harrison EM, Green CA, Hardwick HE, Pius R, Norman L, investigators IC et al Features of 20 133 UK patients in hospital with covid-19 using the ISARIC WHO Clinical Characterisation Protocol: prospective observational cohort study. BMJ (Clinical Research ed.) 2020;369:m1985. 10.1136/bmj.m1985 PMC7243036 32444460

[14] Wu Z , McGoogan JM. Characteristics of and important lessons from the coronavirus disease 2019 (COVID-19) outbreak in China: summary of a report of 72314 cases from the Chinese Center for Disease Control and Prevention. JAMA 2020;323:1239–1242. 32091533 10.1001/jama.2020.2648

[15] Puntmann VO , Carerj ML, Wieters I, Fahim M, Arendt C, Hoffmann J et al Outcomes of cardiovascular magnetic resonance imaging in patients recently recovered from coronavirus disease 2019 (COVID-19). JAMA Cardiol 2020;5:1265–1273. 32730619 10.1001/jamacardio.2020.3557 PMC7385689

[16] Xu Z , Shi L, Wang Y, Zhang J, Huang L, Zhang C et al Pathological findings of COVID-19 associated with acute respiratory distress syndrome. Lancet Respir Med 2020;8:420–422. 32085846 10.1016/S2213-2600(20)30076-X PMC7164771

[17] Liberati A , Altman DG, Tetzlaff J, Mulrow C, Gøtzsche PC, Ioannidis JP, Clarke M et al The PRISMA statement for reporting systematic reviews and meta-analyses of studies that evaluate healthcare interventions: explanation and elaboration. BMJ 2009;339:b2700. 19622552 10.1136/bmj.b2700 PMC2714672

[18] Database of Abstracts of Reviews of Effects (DARE): Quality-assessed Reviews. York (UK): Centre for Reviews and Dissemination (UK); 1995. https://www.ncbi.nlm.nih.gov/books/NBK285222/ (last accessed 7 May 2021).

[19] Shea BJ , Reeves BC, Wells G, Thuku M, Hamel C, Moran J et al AMSTAR 2: a critical appraisal tool for systematic reviews that include randomised or non-randomised studies of healthcare interventions, or both. BMJ 2017;358:j4008. 28935701 10.1136/bmj.j4008 PMC5833365

[20] Biswas M , Rahaman S, Biswas TK, Haque Z, Ibrahim B. *Effects of Sex, Age and Comorbidities on the Risk of Infection and Death Associated with COVID-19: A Meta-Analysis of 47807 Confirmed Cases*. SSRN 2020. Preprint available at SSRN 10.2139/ssrn.3566146.

[21] Chang R , Elhusseiny K, Yeh Y, Sun W. *COVID-19 ICU and Mechanical Ventilation Patient Characteristics and Outcomes - A Systematic Review and Meta-analysis*. ResearchSquare 2020. Preprint available at Research Square 10.212.03/rs.3.rs-66766/v1. PMC7877631 33571301

[22] De Lorenzo A , Kasal DA, Tura BR, Lamas CC, Rey HC. Acute cardiac injury in patients with COVID-19. Am J Cardiovasc Dis 2020;10:28–33. 32685261 PMC7364273

[23] Fang X , Li S, Yu H, Wang P, Zhang Y, Chen Z et al Epidemiological, comorbidity factors with severity and prognosis of COVID-19: a systematic review and meta-analysis. Aging 2020;12:12493–12503. 32658868 10.18632/aging.103579 PMC7377860

[24] Figliozzi S , Masci PG, Ahmadi N, Tondi L, Koutli E, Aimo A et al Predictors of adverse prognosis in COVID-19: a systematic review and meta-analysis. Eur J Clin Invest 2020;50:e13362. 32726868 10.1111/eci.13362

[25] Florez-Perdomo WA , Serrato-Vargas SA, Bosque-Varela P, Moscote-Salazar LR, Joaquim AF, Agrawal A et al Relationship between the history of cerebrovascular disease and mortality in COVID-19 patients: a systematic review and meta-analysis. Clin Neurol Neurosurg 2020;197:106183. 32919240 10.1016/j.clineuro.2020.106183 PMC7446719

[26] Fu L , Wang B, Yuan T, Chen X, Ao Y, Fitzpatrick T et al Clinical characteristics of coronavirus disease 2019 (COVID-19) in China: a systematic review and meta-analysis. J Infect 2020;80:656–665. 32283155 10.1016/j.jinf.2020.03.041 PMC7151416

[27] Gu ZC , Zhang C, Kong LC, Shen L, Li Z, Ge H et al Incidence of myocardial injury in coronavirus disease 2019 (COVID-19): a pooled analysis of 7,679 patients from 53 studies. Cardiovasc Diagn Ther 2020;10:667–677. 32968623 10.21037/cdt-20-535 PMC7487385

[28] Gulsen A , Arpinar Yigitbas B, Uslu B, Droemann D, Kilinc O, The effect of smoking on COVID-19 symptom severity: systematic review and meta-analysis. medRxiv 2020. Preprint available at medRxiv 10.1101/2020.08.15.20102699. PMC7499286 32963831

[29] Hamam O , Goda A, Eldalal M, Ussama A, Fahmy M, Elyamany K, Ikram W, Mahdy A, Bhandari R, Asal H, Egbe A, Cardiac arrhythmias in patients with COVID-19: a systematic review and meta-analysis. medRxiv 2020. Preprint available at medRXiv 10.1101/2020.10.09.20209379.

[30] Hu Y , Sun J, Dai Z, Deng H, Li X, Huang Q et al Prevalence and severity of corona virus disease 2019 (COVID-19): a systematic review and meta-analysis. J Clin Virol 2020;127:104371. 32315817 10.1016/j.jcv.2020.104371 PMC7195434

[31] Islam MS , Barek MA, Aziz MA, Aka TD, Jakaria M. Association of age, sex, comorbidities, and clinical symptoms with the severity and mortality of COVID-19 cases: a meta-analysis with 85 studies and 67299 cases. medRxiv 2020. Preprint available at medRXiv 10.1101/2020.05.23.20110965. PMC7737518 33344791

[32] Lu L , Zhong W, Bian Z, Li Z, Zhang K, Liang B et al A Comparison of mortality-related risk factors of COVID-19, SARS, and MERS: a systematic review and meta-analysis. J Infect 2020;81:e18–e25. 10.1016/j.jinf.2020.07.002 PMC7334925 32634459

[33] Luo L , Fu M, Li Y, Hu S, Luo J, Chen Z et al The potential association between common comorbidities and severity and mortality of coronavirus disease 2019: a pooled analysis. Clin Cardiol 2020;43:1478–1493. 33026120 10.1002/clc.23465 PMC7675427

[34] Mantovani A , Byrne CD, Zheng MH, Targher G. Diabetes as a risk factor for greater COVID-19 severity and in-hospital death: a meta-analysis of observational studies. Nutr Metab Cardiovasc Dis 2020;30:1236–1248. 32571616 10.1016/j.numecd.2020.05.014 PMC7258796

[35] Matsushita K , Ding N, Kou M, Hu X, Chen M, Gao Y et al The relationship of COVID-19 severity with cardiovascular disease and its traditional risk factors: a systematic review and meta-analysis. medRxiv 2020. Preprint available at medRXiv 10.1101/2020.04.05.20054155. PMC7546112 33150129

[36] Momtazmanesh S , Shobeiri P, Hanaei S, Mahmoud-Elsayed H, Dalvi B, Malakan Rad E. Cardiovascular disease in COVID-19: a systematic review and meta-analysis of 10,898 patients and proposal of a triage risk stratification tool. Egypt Heart J 2020;72:41. 32661796 10.1186/s43044-020-00075-z PMC7356124

[37] Nasiri MJ , Haddadi S, Tahvildari A, Farsi Y, Arbabi M, Hasanzadeh S et al COVID-19 clinical characteristics, and sex-specific risk of mortality: systematic review and meta-analysis. medRxiv 2020. Preprint available at medRXiv 10.1101/2020.03.24.2004.2903. PMC7385184 32793620

[38] Villalobos N , Ott J, Klett-Tammen C, Bockey A, Vanella P, Krause G et al Quantification of the association between predisposing health conditions, demographic, and behavioural factors with hospitalisation, intensive care unit admission, and death from COVID-19: a systematic review and meta-analysis. medRxiv 2020. Preprint available at medRXiv 10.1101/2020.07.30.20165050.

[39] Noor FM , Islam MM. Prevalence and associated risk factors of mortality among COVID-19 patients: a meta-analysis. J Community Health 2020;45:1270–1282. 32918645 10.1007/s10900-020-00920-x PMC7486583

[40] Palaiodimos L , Chamorro-Pareja N, Karamanis D, Li W, Zavras PD, Mathias P et al Diabetes is associated with increased risk for in-hospital mortality in patients with COVID-19: a systematic review and meta-analysis comprising 18,506 patients. medRxiv 2020. Preprint available at medRXiv 10.1101/2020.05.26.20113811. PMC7595056 33123973

[41] Patanavanich R , Glantz SA. Smoking is associated with worse outcomes of COVID-19 particularly among younger adults: a systematic review and meta-analysis. medRxiv 2020. Preprint available at medRXiv 10.1101/2020.09.22.20199802 PMC8366155 34399729

[42] Sabatino J , De Rosa S, Di Salvo G, Indolfi C. Impact of cardiovascular risk profile on COVID-19 outcome. A meta-analysis. PLoS One 2020;15:e0237131. 10.1371/journal.pone.0237131 PMC7428172 32797054

[43] Sinclair JE , Zhu Y, Xu G, Ma W, Shi H, Ma K-L et al The role of pre-existing chronic disease in cardiac complications from SARS-CoV-2 infection: a systematic review and meta-analysis. medRxiv 2020. Preprint available at medRXiv 10.1101/2020.06.21.2013.6622.

[44] Ssentongo P , Ssentongo AE, Heilbrunn ES, Ba DM, Chinchilli VM. Association of cardiovascular disease and 10 other pre-existing comorbidities with COVID-19 mortality: a systematic review and meta-analysis. PLoS One 2020;15:e0238215. 32845926 10.1371/journal.pone.0238215 PMC7449476

[45] Tamara A , Tahapary DL. Obesity as a predictor for a poor prognosis of COVID-19: a systematic review. Diabetes Metab Syndr 2020;14:655–659. 32438328 10.1016/j.dsx.2020.05.020 PMC7217103

[46] Wu X , Liu L, Jiao J, Yang L, Zhu B, Li X. Characterization of clinical, laboratory and imaging factors related to mild vs. severe Covid-19 infection: a systematic review and meta-analysis. Ann Med 2020;52:1–21. 32755287 10.1080/07853890.2020.1802061 PMC7877997

[47] Wu ZH , Tang Y, Cheng Q. Diabetes increases the mortality of patients with COVID-19: a meta-analysis. Acta Diabetol 2020;58:139–144. 32583078 10.1007/s00592-020-01546-0 PMC7311595

[48] Youssef M , H Hussein M, Attia AS, M Elshazli R, Omar M, Zora G et al COVID-19 and liver dysfunction: a systematic review and meta-analysis of retrospective studies. J Med Virol 2020;92:1825–1833. 32445489 10.1002/jmv.26055 PMC7283797

[49] Yu JN , Wu BB, Yang J, Lei XL, Shen WQ. Cardio-cerebrovascular disease is associated with severity and mortality of COVID-19: a systematic review and meta-analysis. Biol Res Nurs 2020;23:258–269. 32851851 10.1177/1099800420951984 PMC7481655

[50] Zhang C , Shen L, Le KJ, Pan MM, Kong LC, Gu ZC et al Incidence of venous thromboembolism in hospitalized coronavirus disease 2019 patients: a systematic review and meta-analysis. Front Cardiovasc Med 2020;7:151. 32850990 10.3389/fcvm.2020.00151 PMC7423832

[51] Reddy RK , Charles WN, Sklavounos A, Dutt A, Seed PT, Khajuria A. The effect of smoking on COVID-19 severity: a systematic review and meta-analysis. J Med Virol 2021;93:1045–1056. 32749705 10.1002/jmv.26389 PMC7436545

[52] Madjid M , Safavi-Naeini P, Solomon SD, Vardeny O. Potential effects of coronaviruses on the cardiovascular system: a review. JAMA Cardiol 2020;5:831–840. 32219363 10.1001/jamacardio.2020.1286

[53] Madjid M , Miller CC, Zarubaev VV, Marinich IG, Kiselev OI, Lobzin YV et al Influenza epidemics and acute respiratory disease activity are associated with a surge in autopsy-confirmed coronary heart disease death: results from 8 years of autopsies in 34,892 subjects. Eur Heart J 2007;28:1205–1210. 17440221 10.1093/eurheartj/ehm035 PMC7108465

[54] Banerjee A , Chen S, Pasea L, Lai AG, Katsoulis M, Denaxas S et al Excess deaths in people with cardiovascular diseases during the COVID-19 pandemic. Eur J Prev Cardiol 2021. Online ahead of print doi:10.1093/eurjpc/zwaa155 10.1093/eurjpc/zwaa155 PMC7928969 33611594

[55] Smeeth L , Thomas SL, Hall AJ, Hubbard R, Farrington P, Vallance P. Risk of myocardial infarction and stroke after acute infection or vaccination. N Engl J Med 2004;351:2611–2618. 15602021 10.1056/NEJMoa041747

[56] Colling ME , Kanthi Y. COVID-19-associated coagulopathy: an exploration of mechanisms. Vasc Med 2020;25:471–478. 32558620 10.1177/1358863X20932640 PMC7306998

[57] Driggin E , Madhavan MV, Bikdeli B, Chuich T, Laracy J, Biondi-Zoccai G et al Cardiovascular considerations for patients, health care workers, and health systems during the COVID-19 pandemic. J Am Coll Cardiol 2020;75:2352–2371. 32201335 10.1016/j.jacc.2020.03.031 PMC7198856

[58] Oelsner EC , Balte PP, Bhatt SP, Cassano PA, Couper D, Folsom AR et al Lung function decline in former smokers and low-intensity current smokers: a secondary data analysis of the NHLBI Pooled Cohorts Study. Lancet Respir Med 2020;8:34–44. 31606435 10.1016/S2213-2600(19)30276-0 PMC7261004

[59] Warkentin MT , Lam S, Hung RJ. Determinants of impaired lung function and lung cancer prediction among never-smokers in the UK Biobank cohort. EBioMedicine 2019;47:58–64. 31495719 10.1016/j.ebiom.2019.08.058 PMC6796498

