# Supplementary material for: Cardiovascular risk factors, cardiovascular disease, and COVID-19: an umbrella review of systematic reviews
Source: Eur Heart J Qual Care Clin Outcomes. 2021 Jun 9;7(4):330–9. doi: 10.1093/ehjqcco/qcab029 (PMC8294691; doi:10.1093/ehjqcco/qcab029)
Supplement: qcab029_Supplementary_Data [file qcab029_supplementary_data.pdf]

Table S1. Medline Search Strategy.

#	Searches
1	exp Comorbidity/
2	comorbidit*.tw.
3	exp Cardiovascular Diseases/
4	exp Cardiology/
5	(((heart or cardiovascular) adj (disease* or illness* or anomal* or infection* or abnormalit*)) or CVD or cardio* or cardiac*).tw.
6	1 or 2 or 3 or 4 or 5
7	exp Coronavirus/
8	exp Coronavirus Infections/
9	((corona* or corono*) adj1 (virus* or viral* or virinae*)).tw.
10	(coronavirus* or coronovirus* or coronavirinae* or Coronavirus* or Coronovirus*).tw.
11	(Wuhan* or Hubei* or Huanan or "2019-nCoV" or 2019nCoV or nCoV2019 or "nCoV-2019").tw.
12	(COVID-19 or COVID19).tw.
13	(SARS-CoV-2 or SARSCoV-2 or SARSCoV2 or SARS-CoV2 or SARSCov19 or SARS-Cov19 or SARSCov-19 or SARS-Cov-19).tw.
14	(((respiratory* adj2 (symptom* or disease* or illness* or condition*)) or "seafood market*" or "food market*") adj10 (Wuhan* or Hubei* or China* or Chinese* or Huanan*)).tw.
15	((outbreak* or wildlife* or pandemic* or epidemic*) adj1 (China* or Chinese* or Huanan*)).tw.
16	"severe acute respiratory syndrome*".tw.
17	("long covid" or "long covid-19" or "long covid19" or "long coronav*" or "post acute covid" or "post acute coronav*").tw.
18	7 or 8 or 9 or 10 or 11 or 12 or 13 or 14 or 15 or 16 or 17
19	exp Smoking/
20	smoking.tw.
21	exp Hypertension/
22	hypertens*.tw.
23	"high blood pressure".tw.
24	exp Obesity/
25	obes*.tw.
26	exp Sedentary Behavior/
27	("sedentary behavio?r*" or "physical inactiv*").tw.
28	exp Alcohol-Induced Disorders/
29	alcohol.tw.
30	exp Diet/
31	diet*.tw.
32	exp Cholesterol/
33	cholesterol.tw.
34	19 or 20 or 21 or 22 or 23 or 24 or 25 or 26 or 27 or 28 or 29 or 30 or 31 or 32 or 33
35	exp Atrial Fibrillation/
36	"atrial fibrillation".tw.
37	exp Hyperlipoproteinemia Type II/
38	hyperlipoproteinemia*.tw.
39	"familial hypercholesterolemia".tw.
40	exp Dementia/
41	dementia.tw.
42	exp Hyperglycemia/

43	hyperglycemia.tw.
44	exp Prediabetic State/
45	("prediabetic state" or "pre diabetic state" or "pre diabet*").tw.
46	exp Renal Insufficiency/
47	exp Kidney Diseases/
48	((kidney or renal) adj diseas*).tw.
49	exp Liver Diseases/
50	liver disease*.tw.
51	exp Fibrosis/
52	fibrosis.tw.
53	cirrhosis*.tw.
54	exp Stroke/
55	stroke*.tw.
56	35 or 36 or 37 or 38 or 39 or 40 or 41 or 42 or 43 or 44 or 45 or 46 or 47 or 48 or 49 or 50 or 51 or 52 or 53 or 54 or 55
57	(((comprehensive* or systematic*) adj3 (bibliographic* or review* or literature)) or (meta-analy* or metaanaly* or "research synthesis" or ((information or data) adj3 synthesis) or (data adj2 extract*))).ti,ab. or (cinahl or (cochrane adj3 trial*) or embase or medline or psyclit or (psycinfo not "psycinfo database") or pubmed or scopus or "sociological abstracts" or "web of science").ab. or "cochrane database of systematic reviews".jn. or ((review adj5 (rationale or evidence)).ti,ab. and review.pt.) or meta-analysis as topic/ or Meta-Analysis.pt.
58	exp "Systematic Review"/
59	57 or 58
60	6 and 18 and 34 and 56 and 59
61	limit 60 to (english language and humans and yr="2020 - 2021")

Table S2. AMSTAR 2 ratings for the included reviews.

Study first and second author	AMSTAR 2 Rating	1		2			3		4			5		6		7			8			9			10		11		12		13		14		15		16	
		Y	N	Y	PY	N	Y	N	Y	PY	N	Y	N	Y	N	Y	PY	N	Y	PY	N	Y	PY	N	Y	N	Y	N	Y	N	Y	N	Y	N	Y	N	Y	N
Aggarwal, Cheruiyot[1]	Low quality		X	X			X			X		X		X			X				X	X				X	X		X			X		X	X		X	
Almeshari, Alobaidi[2]	Low quality	X		X				X		X		X		X				X		X			X			X	X			X		X		X		X		X
Alqahtani, Oyelade[3]	Low quality	X		X			X			X		X		X				X		X		X				X	X			X		X	X			X	X	
Bajgain, Badal[4]	Critically low quality	X				X		X		X			X		X			X			X			X		X	X			X		X		X		X	X	
Barrera, Shekhar[5]	Low quality	X		X			X				X	X		X				X		X		X				X	X		X		X		X			X	X	
Bennett, Tafuro[6]	Low quality	X				X		X		X		X		X				X		X		X				X		NA		NA		X		X		NA	X	
Bhatia, Pedapati[7]	Critically low quality	X				X	X			X		X			X			X			X			X		X		NA		NA		X		X		NA	X	
Biswas, Rahaman[8]	Moderate quality	X				X		X		X		X			X			X		X		X				X	X		X		X			X	X		X	
Chang, Elhusseiny[9]	Moderate quality	X			X			X		X		X		X				X		X		X				X	X		X		X			X	X		X	
Chen, Gong[10]	Low quality	X				X		X		X		X		X				X		X		X				X	X		X			X	X		X		X	
Chidambaram, Tun[11]	Low quality	X		X			X				X	X		X				X	X			X				X	X		X		X		X		X		X	
De Lorenzo, Kasal[12]	Moderate quality	X				X		X		X		X			X			X			X	X				X	X		X		X		X		X		X	
Fang, Li[13]	Moderate quality	X				X		X		X			X	X				X			X		X			X	X		X		X		X		X		X	
Figliozzi, Masci[14]	Moderate quality	X		X			X		X				X		X			X	X				X			X	X		X		X			X	X		X	
Flook, Jackson[15]	Critically low quality	X		X			X			X		X		X				X			X			X		X		NA		NA		X		X		NA	X	
Florez-Perdomo, Serrato-Vargas[16]	Moderate quality	X				X	X		X			X			X			X			X	X				X	X		X		X			X	X		X	
Fridman, Bullrich[17]	Critically low quality	X				X		X		X		X			X			X			X			X		X		X	X		X			X	X		X	
Fu, Wang[18]	Moderate quality	X				X		X	X			X		X				X		X		X			X		X		X		X		X		X		X	

Study first and second author	AMSTAR 2 Rating	1		2			3		4			5		6		7			8			9			10		11		12		13		14		15		16	
		Y	N	Y	PY	N	Y	N	Y	PY	N	Y	N	Y	N	Y	PY	N	Y	PY	N	Y	PY	N	Y	N	Y	N	Y	N	Y	N	Y	N	Y	N	Y	N
Gu, Zhang[19]	Moderate quality	X				X	X			X		X		X				X		X		X				X	X		X		X		X		X		X	
Gulsen, Yigitbas[20]	Moderate quality	X		X			X			X		X		X				X		X		X				X	X		X		X		X		X		X	
Hamam, Goda[21]	Moderate quality	X				X		X		X		X		X				X		X			X			X	X		X		X		X			X	X	
Hammoud, Bendari[22]	Critically low quality	X				X		X		X		X			X			X			X			X		N		NA		NA		X		X		NA	X	
Han, Diao[23]	Critically low quality	X		X			X			X			X	X				X		X			X			X		X		X		X	X		X		X	
Hessami, Shamshirian[24]	Low quality	X			X		X			X		X		X				X		X			X			X		X	X		X			X	X		X	
Hu, Sun[25]	Moderate quality	X				X	X			X			X	X				X			X	X				X	X		X		X		X		X		X	
Islam, Barek[26]	Moderate quality	X				X		X		X		X		X				X			X	X				X	X		X		X			X	X		X	
Izcovich, Ragusa[27]	Low quality	X		X			X		X			X		X				X			X	X				X	X		X			X		X		X	X	
Jain, Yuan[28]	Low quality	X		X			X		X			X		X				X		X		X				X	X			X		X		X		X	X	
Khan, Khan[29]	Low quality	X				X	X		X			X		X				X		X		X				X	X		X			X	X		X		X	
Kumar, Arora, Clinical Features[30]	Critically low quality	X				X	X				X		X		X			X		X		X				X	X			X		X		X		X	X	
Kumar, Arora, Diabetes[31]	Low quality	X		X			X				X	X			X			X		X		X				X	X		X		X		X		X		X	
Li, Guan[32]	Low quality	X				X		X			X	X		X				X		X		X				X	X		X		X		X		X		X	
Li, He[33]	Low quality	X				X	X			X			X	X				X			X	X				X	X		X			X	X			X	X	
Li, Huang[34]	Low quality	X		X			X			X		X			X			X			X	X				X	X		X			X	X		X			X
Liu, Chen[35]	Low quality	X				X		X	X			X		X				X		X		X				X	X		X			X		X	X		X	
Liu, Zhang[36]	Critically low quality	X			X		X			X		X		X				X		X			X			X		X		X		X		X	X		X	
Lu, Zhong[37]	Moderate quality	X		X				X	X			X		X				X			X	X				X	X		X		X		X		X		X	

Study first and second author	AMSTAR 2 Rating	1		2			3		4			5		6		7			8			9			10		11		12		13		14		15		16	
		Y	N	Y	PY	N	Y	N	Y	PY	N	Y	N	Y	N	Y	PY	N	Y	PY	N	Y	PY	N	Y	N	Y	N	Y	N	Y	N	Y	N	Y	N	Y	N
Luo, Fu[38]	Moderate quality	X				X		X	X			X		X				X		X		X				X	X		X		X		X		X			X
Ma, Gu[39]	Low quality	X		X			X				X	X		X				X	X			X				X	X		X		X		X		X		X	
Mantovani, Byrne[40]	Moderate quality	X				X	X			X		X			X			X		X		X				X	X		X		X		X		X		X	
Mao, Lin[41]	Critically low quality	X		X			X				X		X	X				X	X				X			X		X		X		X		X	X			
Matsushita, Ding[42]	Moderate quality	X				X	X			X		X		X				X		X		X				X	X		X		X		X		X			X
Momtazmanesh, Shobeiri[43]	Moderate quality	X				X		X		X		X		X				X	X			X				X	X		X		X			X	X		X	
Moula, Micali[44]	Low quality	X				X		X			X		X		X			X		X		X				X	X		X		X		X		X		X	
Nannoni, de Groot[45]	Critically low quality	X		X			X			X		X			X			X			X			X		X		X	X		X			X		X	X	
Nasiri, Haddadi[46]	Moderate quality	X				X		X	X			X		X				X			X	X				X	X		X		X			X	X		X	
Noor, Islam[47]	Moderate quality	X				X		X	X			X		X				X			X	X				X	X		X		X			X	X		X	
Palaiodimos, Chamorro-Pareja[48]	Moderate quality	X				X		X		X		X		X				X		X		X				X	X		X		X		X		X		X	
Parohan, Yaghoubi[49]	Critically low quality	X				X		X		X			X	X				X		X		X				X	X		X		X			X	X		X	
Parveen, Sehar[50]	Critically low quality	X				X		X		X		X		X				X			X	X				X	X			X		X		X		X	X	
Patanavanich, Glantz[51]	Moderate quality	X		X			X			X			X	X				X		X		X				X	X		X		X		X		X		X	
Patel, Malik, Shah[52]	Critically low quality	X		X			X				X	X		X				X		X		X				X	X		X		X		X			X	X	
Patel, Malik, Usman[53]	Critically low quality	X		X			X		X			X		X				X		X		X				X		X		X		X	X			X	X	
Porto, Iamonti[54]	Critically low quality	X			X		X				X	X			X			X		X			X			X		X	X		X			X		X	X	
Reddy, Charles[55]	High quality	X		X			X			X		X		X				X	X			X			X		X		X		X		X		X		X	
Rhim, Park[56]	Low quality	X		X			X			X		X			X			X		X		X				X	X			X		X	X		X		X	

Study first and second author	AMSTAR 2 Rating	1		2			3		4			5		6		7			8			9			10		11		12		13		14		15		16	
		Y	N	Y	PY	N	Y	N	Y	PY	N	Y	N	Y	N	Y	PY	N	Y	PY	N	Y	PY	N	Y	N	Y	N	Y	N	Y	N	Y	N	Y	N	Y	N
Roncon, Zuin[57]	Critically low quality	X			X		X				X	X		X				X		X			X			X		X	X		X		X		X		X	
Sabatino, De Rosa[58]	Moderate quality	X		X			X			X		X		X				X	X			X				X	X		X		X		X		X		X	
Sales-Peres, Azevedo-Silva[59]	Critically low quality	X		X			X				X	X			X			X		X			X			X		X	X		X		X		X		X	
Sepandi, Taghdir[60]	Critically low quality	X		X			X				X		X	X				X		X			X			X		X	X		X			X	X		X	
Shafi, Shaikh[61]	Low quality	X			X		X			X		X		X				X		X			X			X		NA		NA		X		X		NA	X	
Shao, Shang[62]	Low quality	X			X		X			X			X		X			X		X			X			X		X	X		X		X		X		X	
Shi, Wang[63]	Low quality	X		X			X			X			X	X				X	X				X			X	X			X		X	X		X		X	
Sinclair, Zhu[64]	Moderate quality	X			X		X			X			X	X				X		X			X			X	X		X		X		X			X	X	
Sreenivasan, Khan[65]	Critically low quality	X			X		X			X		X		X		X			X				X			X		X		X		X		X		X	X	
Ssentongo, Ssentongo[66]	Moderate quality	X		X			X			X		X		X				X	X			X				X	X		X		X		X		X		X	
Tabrizi, Lankarani[67]	Critically low quality	X		X			X				X	X		X				X		X		X				X		X		X		X	X		X		X	
Tamara, Tahapary[68]	Moderate quality	X			X		X			X			X		X			X		X			X			X		NA		NA	X		X			NA	X	
Taylor, Hofmeyr[69]	Critically low quality	X			X		X				X	X		X				X		X			X			X		X		X		X	X			X	X	
Tian, Jiang[70]	Critically low quality	X			X		X			X		X		X				X	X				X			X		X		X		X		X		X		
Villalobos, Ott[71]	Moderate quality	X		X			X			X			X	X				X			X	X				X	X		X		X		X		X		X	
Wang, Deng[72]	Critically low quality	X		X			X				X	X		X				X	X			X				X		X	X		X		X		X		X	
Wang, Li[73]	Critically low quality	X		X			X			X		X		X				X		X			X			X		X		X		X	X		X		X	
Wu, Liu[74]	Moderate quality	X		X			X			X			X	X				X	X			X				X	X		X		X		X		X		X	
Wu, Tang[75]	Moderate quality	X		X			X			X			X		X			X		X			X			X	X		X		X		X		X		X	

Study first and second author	AMSTAR 2 Rating	1		2			3		4			5		6		7			8			9			10		11		12		13		14		15		16	
		Y	N	Y	PY	N	Y	N	Y	PY	N	Y	N	Y	N	Y	PY	N	Y	PY	N	Y	PY	N	Y	N	Y	N	Y	N	Y	N	Y	N	Y	N	Y	N
Wu, Zuo[76]	Critically low quality	X			X		X			X		X		X				X		X		X				X		X		X		X		X	X		X	
Xu, Mao[77]	Critically low quality	X			X		X			X			X	X				X	X				X			X		X		X		X		X	X		X	
Youssef, Hussein[78]	Moderate quality	X		X			X			X		X		X				X		X		X				X	X		X		X		X		X		X	
Yu, Wu[79]	Moderate quality	X		X			X			X		X		X				X		X		X				X	X		X		X		X		X		X	
Zhang, Shen[80]	Moderate quality	X		X			X			X		X			X			X	X			X				X	X		X		X		X		X		X	
Zhang, Wu[81]	Critically low quality	X			X		X				X	X			X			X		X			X			X	X			X		X	X		X		X X	
Zhao, Meng[82]	Critically low quality	X		X			X				X	X		X				X		X			X			X	X			X		X	X		X		X X	
Zheng, Peng[83]	Low quality	X		X			X			X			X	X				X		X			X			X	X			X		X	X			X	X	
Zuin, Rigatelli[84]	Low quality	X		X			X			X		X		X				X		X			X			X	X			X		X	X		X		X	

NA: not applicable (meta-analysis not performed to score this category)

Table S3. Characteristics and results of all included reviews.

**Study characteristics**<br><br>**First and second author surname**<br><br>**Search dates**<br><br>**N=studies (n=patients)**<br>**Primary study countries**<br><br>**Study designs of included primary studies**<br>**Quality assessment of primary studies**<br><br>**AMSTAR2 grade**	**Exposures examined**<br><br>**COVID-19 patients and cardiovascular condition(s) reported in primary data**	**Outcomes examined**	**Systematic review/ meta-analysis results**<br><br>**Results of meta-analyses are presented unless otherwise stated, such as narrative review findings from a systematic review without meta-analysis.**
Aggarwal, Cheruiyot[1]<br><br>November 1, 2019 to April 20, 2020<br><br>N=18 studies (4,858 patients)<br>16 China, 2 US<br><br>Case-control/cohort<br>Newcastle-Ottawa Scale, range 6-9<br><br>AMSTAR2: Low quality	CVD<br>defined as any cardiac pathology with the exception of hypertension	Severe COVID*<br>Mortality<br>Mortality in severe disease<br><br>*Composite outcome of (1) Respiratory distress, respiratory rate $\geq$30 per minute; (2) Oxygen saturation at rest $\leq$93%; (3) Partial pressure of oxygen in arterial blood/fraction of inspired oxygen $\leq$300 mmhg; (4) Patients requiring mechanical ventilation/vital life support/intensive care unit admission; (5) Death.	OR (95% CI)<br><br>CVD and Severe COVID<br>3.14 (2.32-4.24) I^2=0%<br><br>CVD and mortality<br>11.08 (2.59-47.32) I^2=55%<br><br>CVD and mortality in severe disease<br>1.72 (0.97-3.06) I^2=0%<br><br>Meta-regression of odds of severe disease with CVD:<br>The age of patients in the severe group had no significant influence (P=0.34).<br>As the percentage of women in the severe group increased, so did the odds ratio of severe disease and CVD association (P=0.02).
Almeshari, Alobaidi[2]<br><br>December 1, 2019 to April 23, 2020<br><br>N=16 studies; 2 cardiac injury (9,988 patients; 603 cardiac injury)<br>12 China, 3 USA, 1 Italy<br><br>Case-control/Cohort<br>NIH quality assessment tool: Good quality<br><br>AMSTAR2: Low quality	Cardiac injury	Mechanical ventilation<br>Mortality	Systematic review findings:<br><br>One study demonstrated 25% patients required mechanical ventilation (n=52).<br><br>In one study, 60% of patients with elevated Troponin required mechanical ventilation compared to 10% with normal Troponin.<br><br>In one study (n=416), patients with cardiac injury 22% (18/82) required mechanical ventilation compared to 4.2% in those without cardiac injury (14/334).
Alqahtani, Oyelade[3]<br><br>Inception to March 24, 2020	Current/ex-smoker	Severe COVID<br>(those who were admitted to ICU, had severe, oxygenation,	RR (95% CI)<br><br>Current smoking vs ex/never smoked<br>1.45 (1.03–2.04) I^2=92%

N=15 studies; 8 smoking (2,473 patients; 221 smoking studies)<br>14 China, 1 USA<br><br>Case-control/cohort, case-series,<br>Modified Newcastle-Ottawa Scale, range: 0.4 to 2.7 (9 studies >2, indicating low risk of bias)<br><br>AMSTAR2: Low quality		needed mechanical ventilation or death)	
Bajgain, Badal[4]<br><br>Inception to May 15, 2020.<br><br>N=27 studies (22,753 patients)<br>18 China, 2 South Korea, 2 Italy, 2 USA, 1 Mexico, 1 UK, 1 Iran<br><br>Study design NR<br>Newcastle-Ottawa Scale, range 6-10<br><br>AMSTAR2: Critically low quality	Hypertension	Mortality	OR (95% CI)<br><br>Hypertension<br>1.65 (1.01-1.85)
Barrera, Shekhar[5]<br><br>December 1, 2019 to April 6, 2020<br><br>N=65 studies (15,794 patients)<br>46 China, 5 USA, 3 Singapore, 2 Italy, 2 Republic of Korea, 2 Hong Kong, 1 Australia, 1 Bolivia, 1 France, 1 Iran, 1 Japan<br><br>Case-control/cohort, case-series<br>GRADE, 18 low risk of bias, 3 some concerns, 44 high risk of bias. Overall confidence was low.<br><br>AMSTAR2: Low quality	Hypertension<br>Diabetes mellitus<br>Hypertension and diabetes mellitus	Severe COVID*<br>Mortality<br>ICU admission<br><br>*ICU admission or mortality	RR (95% CI)<br><br>Diabetes and severe COVID<br>N=6 studies (1,991 patients)<br>1.50 (0.90-2.50) I^2=74%<br><br>Diabetes and ICU admission<br>N=3 studies (8,890 patients)<br>1.96 (1.19-3.22) I^2=80%<br><br>Diabetes and mortality<br>N=4 studies (2,058 patients)<br>2.78 (1.39-5.58) I^2=75%<br><br>Hypertension and severe COVID<br>N=8 studies (2,023 patients)<br>1.48 (0.99-2.23) I^2=69%<br><br>Hypertension and ICU admission<br>N=4 studies (1,737 patients)<br>2.95 (2.18-3.99) I^2=0%<br><br>Hypertension and mortality<br>N=8 studies (3,107 patients)<br>2.39 (1.54-3.73) I^2=66%
Bennett, Tafuro[6]	COVID-19	Acute cardiac injury	Prevalence of acute cardiac injury

January 1, 2019 to April 26, 2020<br><br>N=45 studies (14,358 patients)<br>42 China, 2 USA, 1 Europe<br>Case-control/cohort, cross-sectional<br><br>Newcastle-Ottawa Scale, range 4-6 (out of 7)<br><br>AMSTAR2: Low quality			N studies=4 (1,096 patients)<br>16.2%
Bhatia, Pedapati[7]<br><br>Inception to May 22, 2020<br><br>N=30 studies (115 patients)<br>Primary study origin NR<br><br>Case-reports, case-series, case-control/cohort<br>Oxford Centre for Evidence-based Medicine's Levels of Evidence and Grades of Recommendation<br>"The risk of bias was not assessed systematically but was likely to be high in all studies since most were case reports, case series, and retrospective observational studies."<br><br>AMSTAR2: Critically low quality	For stroke patients:<br>Type of stroke<br>Hypertension<br>Diabetes<br>Smoking<br>Dyslipidaemia<br>CAD	Mortality	OR (95% CI)<br><br>Ischaemic vs. Non-ischaemic stroke<br>N=73 patients<br>1.1 (0.3-4.1)<br><br>NIH stroke scale (NIHSS)<br>N=13 patients<br>1 (0.9-1.2)<br><br>TOAST criteria<br>N=52 patients<br>Large artery disease<br>0.5 (0.1-2.3)<br>Small vessel disease<br>0.16 (0.01-1.9)<br>Cardioembolic<br>1.3 (0.3-4.7)<br>Cryptogenic<br>1.5 (0.3-7.6)<br><br>Hypertension<br>N=72 patients<br>1.5 (0.6-3.9)<br><br>Diabetes<br>N=72 patients<br>2.1 (0.7-6.3)<br><br>Old stroke<br>N=72 patients<br>2.3 (0.2-2.7)<br><br>Smoking<br>N=47 patients<br>6 (1.1-33.9)<br><br>Atrial fibrillation<br>N=72 patients<br>1.1 (0.3-4.9)<br><br>Dyslipidaemia<br>N=65 patients

			3 (0.96-9.7)<br><br>CAD<br>N=72 patients<br>6.4 (0.7-58)
Biswas, Rahaman[8]<br><br>Inception to March 25, 2020<br><br>N=21 studies (47,807 patients)<br>19 China, 1 South Korea, 1 Singapore<br><br>Case-control/cohort, RCT<br>Newcastle-Ottawa Scale, range 5-8<br><br>AMSTAR2: Moderate quality	Hypertension<br>Diabetes mellitus<br>Cardio-cerebrovascular disease<br>Renal disease<br>CHD	Mortality	RR (95% CI)<br><br>Hypertension<br>N=4 studies (44,975 patients)<br>2.63 (2.32–2.98) I^2=0%<br><br>Diabetes mellitus<br>N=5 studies (44,995 patients)<br>3.34 (2.79–4.0) I^2=0%<br><br>Cardio-cerebrovascular disease<br>N=3 studies (44,701 patients)<br>5.06 (4.13–6.20) I^2=0%<br><br>Renal disease<br>N=3 studies (1,189 patients)<br>3.59 (1.49–8.67) I^2=0%<br><br>CHD<br>N=4 studies (1,346 patients)<br>3.84 (1.64–8.99) I^2=54%
Chang, Elhusseiny[9]<br><br>Inception to May 1, 2020<br><br>N=28 studies (12,437 patients)<br>13 China, 9 USA, 2 UK, 1 Mexico, 1 Italy, 1 Spain, 1 France<br><br>Case-control/cohort<br>NIH quality assessment tool, 15 Good, 13 Fair<br><br>AMSTAR2: Moderate quality	Hypertension<br>Diabetes mellitus<br>CVD<br>Smoking<br>Cerebrovascular disease	ICU Mortality	OR (95% CI)<br><br>Hypertension<br>N=6 studies (1,327 patients)<br>2.02 (1.37-2.98) I^2=52%<br><br>Diabetes mellitus<br>N=5 studies (677 patients)<br>1.78 (1.19-2.65) I^2=42%<br><br>CVD<br>N=6 studies (858 patients)<br>2.77 (1.76-4.37) I^2=45%<br><br>Smoking<br>N=3 studies (318 patients)<br>1.19 (0.48-2.92) I^2=42%<br><br>Cerebrovascular diseases<br>N=2 studies (67 patients)<br>3.84 (0.48-30.89) I^2=17%
Chen, Gong[10]<br><br>Inception to March 6, 2020<br><br>N=9 studies (1,936 patients)<br>9 China<br><br>Case-control/cohort	Hypertension<br>Diabetes mellitus<br>CHD	Severe COVID (severe vs. Non-severe; or intensive care unit (ICU) vs. Non-ICU; or progression and improvement/ stabilization)	OR (95% CI)<br><br>Hypertension<br>N=9 studies (1,936 patients)<br>2.30 (1.76-3.00) I^2=50%<br><br>Diabetes mellitus<br>N=9 studies (1,936 patients)

Newcastle-Ottawa Scale, range 6-7<br><br>AMSTAR2: Low quality			2.67 (1.91-3.74) I^2=42%<br><br>CHD<br>N=6 studies (1,720 patients)<br>2.85 (1.68-4.84) I^2=0%
Chidambaram, Tun[11]<br><br>Inception to May 7, 2020<br><br>N=109 studies (20,296 patients in the comparison of died and survived, 17,992 in the comparison of severe and non-severe)<br>Comparison of died and survived:<br>32 China, 6 USA, 2 Spain, 1 UK, 1 Italy<br>1 Iran, 1 International<br>Comparison of severe vs. Non-severe:<br>71 China, 1 Italy<br><br>Case-control/cohort, cross-sectional, case-series<br>Newcastle-Ottawa Scale, range 4-9 (1 scored 9, 54 scored 8, 39 scored 7, 6 scored 6, 6 scored 5, 3 scored 4)<br><br>AMSTAR2: Low quality	Ever smoker<br>Diabetes mellitus<br>Hypertension<br>CVD<br>Heart failure<br>Cerebrovascular disease<br>Renal disease<br>Chronic liver disease<br>Acute cardiac injury<br>Acute cardiac failure	Mortality<br>Severe COVID*<br><br>*Respiratory rate ≥30 breaths/minute, oxygen saturation ≤93% at rest, arterial oxygen tension (pao2) over inspiratory oxygen fraction (fio2) ratio ≤300 mm Hg or patients with >50% lesions progression within 24 to 48 hours in pulmonary imaging' or having 'evidence of respiratory failure and/or a need for mechanical ventilation, or shock or organ failure that requires intensive care monitoring.	OR (95% CI)<br><br>Ever smoked<br>Mortality: N=7 studies (10,419 patients)<br>1.43 (1.09-1.87) I^2=0%<br>Severe COVID: N=10 studies (4,511 patients)<br>1.51 (1.06-2.14) I^2=62%<br><br>Diabetes mellitus<br>Mortality: N=27 studies (16,263 patients)<br>1.59 (1.41-1.78) I^2=23%<br>Severe COVID: N=36 studies (7,552 patients)<br>2.09 (1.66-2.64) I^2=40%<br><br>Hypertension<br>Mortality: N=26 studies (15,947 patients)<br>1.90 (1.69-2.15) I^2=28%<br>Severe COVID: N=33 studies (7,002 patients)<br>2.63 (2.08-3.33) I^2=64%<br><br>CVD<br>Mortality: N=25 studies (16,576 patients)<br>2.27 (1.88-2.79) I^2=71%<br>Severe COVID: N=31 studies (6,932 patients)<br>2.83 (2.21-3.63) I^2=23%<br><br>Heart failure<br>Mortality: N=5 studies (9,910 patients)<br>2.08 (1.54-2.80) I^2=0%<br>Severe COVID: N=3 studies (558 patients)<br>4.76 (1.34-17.0) I^2=0%<br><br>Cerebrovascular disease<br>Mortality: N=15 studies (2,437 patients)<br>2.63 (1.97-3.51) I^2=75%<br>Severe COVID: N=13 studies (4,246 patients)<br>2.62 (1.76-3.90) I^2=7%<br><br>Renal disease<br>Mortality: N=15 studies (6,556 patients)<br>2.24 (1.78-2.81) I^2=20%<br>Severe COVID: N=14 studies (4,442 patients)

			2.62 (1.46-4.71) I^2=27%<br><br>Chronic liver disease<br>Mortality: N=6 studies (3,672 patients)<br>2.18 (1.40-3.40) I^2=20%<br>Severe COVID: N=17 studies (8,869 patients)<br>1.56 (1.12-2.17) I^2=0%<br><br>Acute cardiac injury<br>Mortality: N=14 studies (2,860 patients)<br>5.42 (3.79-7.77) I^2=86%<br>Severe COVID: N=3 studies (495 patients)<br>3.10 (2.55-3.77) I^2=0%
De Lorenzo, Kasal[12]<br><br>February 4, 2020 OR April 2, 2020<br><br>N=8 studies (1,229 patients)<br>8 China<br><br>Case-control/cohort, case-series<br>Newcastle-Ottawa Scale, 1 Good, 7 Fair<br><br>AMSTAR2: Moderate quality	COVID-19	Prevalence of acute cardiac injury in hospitalised patients	Proportion (95%CI)<br><br>Acute cardiac injury<br>N=8 studies (1,229 patients)<br>0.16 (0.09-0.27) I^2=92%
Fang, Li[13]<br><br>April 5, 2020<br><br>N=69 studies (15,071 patients)<br>67 China, 1 Japan, 1 Singapore<br><br>Case-control/cohort<br>Newcastle-Ottawa Scale, range 5-7<br><br>AMSTAR2: Moderate quality	Comorbidity<br>Hypertension<br>Diabetes mellitus<br>CVD<br>CHD<br>Cerebrovascular disease<br>Renal disease	Severe COVID*<br>Mortality<br>Admission to ICU<br><br>*a. Respiratory distress, respiratory rate ≥ 30/min; b. Oxygen saturation of finger ≤ 93% in resting condition; c. Arterial partial pressure of oxygen (pao2) /oxygen concentration (fio2) ≤ 300 mmhg (1 mmhg = 0.133 kpa); including critical patients a. Respiratory failure requiring mechanical ventilation; b. Shock; c. Concomitant failure of other organs and requirement for ICU.	RR (95% CI)<br><br>Comorbidity<br>Severe COVID: N=16 studies<br>1.72 (1.44-2.06) I^2=83%<br>Mortality: N=8 studies<br>1.68 (1.32-2.12) I^2=89%<br>ICU: N=5 studies<br>1.82 (1.44-2.29) I^2=61%<br><br>Hypertension<br>Severe COVID: N=23 studies<br>2.09 (1.73-2.52) I^2=75%<br>Mortality: N=11 studies<br>1.74 (1.31-2.30) I^2=84%<br>ICU: N=5 studies<br>2.31 (1.99-2.69) I^2=0%<br><br>Diabetes mellitus<br>Severe COVID: N=23 studies<br>1.94 (1.6-2.36) I^2=43%<br>Mortality: N=10 studies<br>1.75 (1.27-2.41) I^2=67%<br>ICU: N=5 studies<br>1.88 (1.10-3.23) I^2=51%<br><br>CVD

			Severe COVID: N=18 studies<br>2.74 (2.03-3.70) I^2=46%<br>Mortality: N=11 studies<br>2.66 (1.60-4.43) I^2=76%<br>ICU: N=5 studies<br>2.83 (1.98-4.05) I^2=0%<br><br>CHD<br>Severe COVID: N=8 studies<br>2.03 (1.39-2.97) I^2=44%<br>Mortality: N=5 studies<br>3.16 (1.45-6.91) I^2=88%<br>ICU: NR<br><br>Cerebrovascular disease<br>Severe COVID: N=12 studies<br>2.77 (1.70-4.52) I^2=40%<br>Mortality: N=6 studies<br>4.55 (2.60-7.94) I^2=0%<br>ICU: N=3 studies<br>4.52 (2.48-8.25) I^2=5%<br><br>Renal disease<br>Severe COVID: N=15 studies<br>2.38 (1.43-3.97) I^2=26%<br>Mortality: N=5 studies<br>7.45 (3.5-15.86) I^2=0%<br>ICU: N=2 studies<br>1.50 (0.37-5.99) I^2=0%
Figliozzi, Masci[14]<br><br>April 24, 2020<br><br>N=49 studies (20,211 patients)<br>Primary study origin NR<br><br>Case-control/cohort, cross-sectional<br>Newcastle-Ottawa Scale, range 7-8<br><br>AMSTAR2: Moderate quality	Diabetes mellitus<br>Hypertension<br>CVD<br>Acute cardiac injury	Composite adverse outcome (mortality, mechanical ventilation, and severe COVID-19)	OR (95% CI)<br><br>Diabetes mellitus<br>N=34 studies (15,953 patients)<br>2.34 (1.64–3.33) I^2=80%<br><br>Hypertension<br>N=35 studies (9,360 patients)<br>2.25 (1.80–2.82) I^2=50%<br><br>CVD<br>N=19 studies (12,717 patients)<br>3.15 (2.26–4.41) I^2=40%<br><br>Acute cardiac injury<br>N=12 studies (2,069 patients)<br>10.58 (5.00–22.40) I^2=59%
Flook, Jackson[15]<br><br>November 1, 2019 to April 29, 2020<br><br>N=33 studies (153,003 patients)<br>29 China, 1 France, 1 Italy, 1 Singapore, 1 UK<br><br>Case-control/cohort	Comorbidity	Mortality	Five (out of 33) studies presented evidence for the presence of any comorbidity being a risk factor for mortality in patients with COVID-19.<br><br>No studies demonstrated evidence against.

The quality of included studies was assessed using an adapted checklist. Included studies were generally too small to detect a 10% increase in risk of disease, disease severity, or mortality. 3 were well power, 26 were descriptive or presented univariable analysis only.<br><br>AMSTAR2: Critically low quality			
Florez-Perdomo, Serrato-Vargas[16]<br><br>Inception to May 2020<br><br>N=7 studies (3,244 patients)<br>6 China, 1 Italy<br><br>Case-control/cohort<br>Newcastle-Ottawa Scale, range 5-6<br><br>AMSTAR2: Moderate quality	Cerebrovascular disease	Mortality	OR (95% CI)<br><br>Cerebrovascular disease<br>N=7 studies (3,244 patients)<br>2.78 (1.42-5.46) I^2=49%
Fridman, Bullrich[17]<br><br>November 1, 2019 to May 29, 2020<br><br>N=10 studies (8,628 patients)<br>Primary data origin NR<br><br>Case-control/cohort, case-series, reports<br>ROBINS-I tool, overall risk of bias was moderate.<br><br>AMSTAR2: Critically low quality	COVID-19	Prevalence of new-onset stroke following COVID-19 diagnosis	Proportion (95% CI)<br><br>Any stroke<br>N=9 studies (3,306 patients)<br>0.02 (0.01-0.04) I^2=84%<br><br>Ischemic stroke<br>N=9 studies (5,322 patients)<br>0.02 (0.01-0.03) I^2=82%
Fu, Wang[18]<br><br>Inception to March 2, 2020<br><br>N=43 studies (3,600 patients)<br>43 China<br><br>Case-control/cohort, case-series<br>NIH quality assessment tool, range 2-7 (9 low risk, 30 moderate, 4 high risk)<br><br>AMSTAR2: Moderate quality	COVID-19	Cardiac failure	Prevalence % (95% CI)<br><br>Cardiac failure<br>N=4 studies (245 patients)<br>6.5 (2.2-12.2) I^2=78%

Gu, Zhang[19]<br><br>April 24, 2020<br><br>N=53 studies (7,679 patients)<br>52 China, 1 USA<br><br>Case-control/cohort, case-series, cross-sectional<br>The methodological quality of included rcts was evaluated according to Cochrane Collaboration Risk of Bias Tool.<br>The methodological quality included observational studies was assessed according to the Newcastle-Ottawa Scale.<br>All 53 studies were rated as relatively good quality, range 5-8.<br><br>AMSTAR2: Moderate quality	COVID-19	Myocardial injury	Incidence (95% CI)<br><br>Myocardial injury<br>N=53 studies (7,679 patients)<br>0.21 (0.17-0.25) I^2=97%<br><br>Myocardial injury in non-survivors<br>N=8 studies (380 patients)<br>0.66 (0.54-0.78) I^2=86%<br><br>RR (95% CI)<br><br>Myocardial injury in severe vs non-severe COVID cases<br>N=29 studies (4,233 patients)<br>5.74 (3.74-8.79) I^2=87%
Gulsen, Yigitbas[20]<br><br>December 2019 to April 15, 2020<br><br>N=16 studies (11,322 patients) in quantitative analyses<br>14 China, 1 USA, 1 CDC report (unknown)<br><br>Case-control/cohort, cross-sectional<br>Newcastle-Ottawa scale, range 5-8 (out of 9)<br><br>AMSTAR2: Moderate quality	Smoking	Prevalence of smokers stratified for severity<br><br>(Studies classified COVID-19 cases broadly as follows: (i) mild to moderate: mild, non-severe, common type, did not require ICU care, and COVID-19 survivors and (ii) severe: severe, critical, required ICU care, and non-survivors.)	OR (95% CI)<br><br>History of smoking<br>Severe vs non-severe COVID<br>N=16 studies (10,797 patients)<br>2.17 (1.37-3.46) I^2=71%<br><br>Severe COVID<br>Current smoker vs non-smoker<br>N=10 studies (9,372 patients)<br>1.51 (1.11-2.05) I^2=49%
Hamam, Goda[21]<br><br>Search dates NR<br><br>N=9 studies (1,445 patients)<br>8 China, 1 USA<br><br>Case-control/cohort<br>Newcastle-Ottawa scale, range 7-8 (out of 9)<br><br>AMSTAR2: Moderate quality	COVID-19	Incidence of arrhythmia	Incidence (95% CI)<br><br>N=9 studies (1,445 patients)<br>0.20 (0.12-0.28) I^2=95%
Hammoud, Bendari[22]	COVID-19 mortality	Histopathologies of the heart	In the 23 articles that described cardiac pathology, the most reported pathology was myocardial hypertrophy (87 cases,

December 2019 to August 15, 2020<br><br>N=50 studies (430 patients)<br>16 USA, 10 China, 6 Germany, 5 Italy, 2 Switzerland, 1 Iran, 1 Finland, 1 Austria, 1 Belgium, 1 Japan, 1 Spain, 1 Netherlands, 1 UK, 1 Romania, 1 Austria, 1 Denmark<br><br>Case-control/cohort, cross-sectional, case report<br>Newcastle-Ottawa Scale (modified)<br>4 Fair (26-50%)<br>26 Good (51-75%)<br>20 Excellent (>76%)<br><br>AMSTAR2: Critically low quality			51%), followed by myocardial fibrosis (85 cases, 50%), coronary small vessel disease (44 cases, 26%) myocardial cell infiltrate (27 cases, 16%), cardiac amyloidosis (10 cases, 6%), and myocardial necrosis (9 cases, 5%).
Han, Diao[23]<br><br>Inception to March 7, 2020<br><br>N=14 studies (1,800 patients)<br>14 China<br><br>Case-control/cohort, case-series<br><br>Newcastle-Ottawa Scale (0-8 points) and CARE statement (0-8 points), all high quality (≥ 5)<br><br>AMSTAR2: Critically low quality	Hypertension<br>CVD<br>Diabetes<br>Cerebrovascular disease<br>Chronic renal disease	Severe COVID (mechanical ventilation, ICU admission or mortality)	OR (95% CI)<br><br>Hypertension<br>N=6 studies (655 patients)<br>2.86 (1.83-4.47) I^2=1%<br><br>CVD<br>N=5 studies (539 patients)<br>3.53 (1.89-6.58) I^2=0%<br><br>Diabetes mellitus<br>N=6 studies (655 patients)<br>3.10 (0.79-12.07) I^2=78%<br><br>Cerebrovascular disease<br>N=2 studies (254 patients)<br>2.53 (0.87-7.41) I^2=0%<br><br>Chronic renal disease<br>N=5 studies (505 patients)<br>2.29 (0.84-6.25) I^2=0%
Hessami, Shamshirian[24]<br><br>Inception to May 27, 2020<br><br>N=56 studies (29,056 patients)<br>Primary study origin NR<br><br>Case-control/cohort, case series<br>Newcastle-Ottawa Scale, all low risk of bias for selection and outcome	Acute cardiac injury<br>Heart failure<br>Arrhythmia<br>Hypertension<br>CVD<br>CHD	Mortality	OR (95% CI)<br><br>Acute cardiac injury<br>N=12 studies<br>13.29 (7.35-24.0) I^2=74%<br><br>Heart failure<br>N=8 studies<br>6.72 (3.34-13.52) I^2=87%<br><br>Arrhythmia<br>N=3 studies<br>2.75 (1.43-5.25) I^2=0%

AMSTAR2: Low quality			Hypertension<br>N=31 studies<br>2.60 (2.11-3.19) I^2=74%<br><br>CVD<br>N=14 studies<br>2.61 (1.89-3.62) I^2=56%<br><br>CHD<br>N=16 studies<br>3.78 (2.42-5.90) I^2=76%<br><br>Prevalence of acute cardiac injury in ICU<br>N=8 studies<br>0.33 (0.24-0.43) I^2=51%
Hu, Sun[25]<br><br>Inception to March 10, 2020<br><br>N=21 studies (47,344 patients)<br>20 China, 1 Singapore<br><br>Case-control/cohort<br>Newcastle-Ottawa Scale, range 5-8 (out of 9)<br><br>AMSTAR2: Moderate quality	COVID-19	Acute cardiac injury<br><br>Severe COVID	Incidence (95% CI)<br><br>Acute cardiac injury<br>N=4 studies<br>0.06 (0.01-0.11) I^2=72%
Islam, Barek[26]<br><br>January 1, 2020, to May 17, 2020<br><br>N=85 studies (67,299 patients)<br>69 China, 8 USA, 6 Italy, 1 South Korea, 1 Iran<br><br>Case-control/cohort<br>Newcastle-Ottawa scale, range 5-8 (out of 9)<br>83 High quality (6-8), 2 Moderate (5)<br><br>AMSTAR2: Moderate quality	Comorbidity<br>Hypertension<br>CVD<br>Diabetes mellitus<br>Cerebrovascular disease<br>Renal disease<br>Liver disease	Mortality	OR (95% CI)<br><br>Any comorbidity<br>N=6 studies (927 patients)<br>3.46 (2.56-4.67) I^2=0%<br><br>Hypertension<br>N=19 studies (47,797 patients)<br>3.16 (2.51-3.97) I^2=44%<br><br>CVD<br>N=20 studies (47,685 patients)<br>4.67 (3.22-6.77) I^2=54%<br><br>Diabetes mellitus<br>N=21 studies (47,864 patients)<br>2.45 (1.82-3.30) I^2=49%<br><br>Cerebrovascular disease<br>N=13 studies (2,183 patients)<br>5.84 (3.63-9.39) I^2=8%<br><br>Renal disease<br>N=9 studies (1,977 patients)<br>5.62 (3.34-9.46) I^2=0%<br><br>Liver disease<br>N=8 studies (1,350 patients)<br>2.81 (1.31-6.01) I^2=0%

Izcovich, Ragusa[27]<br><br>Inception to April 28, 2020<br><br>N=207 studies (75,607 patients)<br>China, USA, Canada, Spain, France, Turkey, Korea, Japan, Italy, Germany, India and Singapore<br><br>Primary study design NR<br>Quality in Prognosis Studies tool (QUIPS)<br>Risk of bias was high across most identified studies. Only 7 were low risk of bias. The remaining presented important limitations in at least one domain or item.<br><br>AMSTAR2: Low quality	Smoking<br>Any chronic condition or comorbidity<br>Cerebrovascular disease<br>Chronic kidney disease (Renal disease)<br>CVD (CHD or Heart failure)<br>Cardiac arrhythmia<br>Arterial hypertension<br>Diabetes mellitus<br>Obesity<br>Dyslipidaemia	Mortality<br>Severe COVID-19*<br><br>*based on primary study definitions	OR (95% CI)<br><br>Current smoker<br>Mortality: N=16 studies (12,025 patients)<br>1.57 (1.19-2.07)<br>Severe COVID-19: N=45 studies (9,147 patients)<br>1.65 (1.25-2.17)<br><br>Comorbidity<br>Mortality: N=16 studies (4,406 patients)<br>3.3 (2.18 to 5)<br>Severe COVID-19: N=40 studies (6,640 patients)<br>3.16 (2.71-3.68)<br><br>Cerebrovascular disease<br>Mortality: N=26 studies (15,294 patients)<br>2.85 (2.02 to 4.01)<br>Severe COVID-19: N=42 studies (11,050 patients)<br>2.67 (1.84-3.87)<br><br>Renal disease<br>Mortality: N=28 studies (23,448 patients)<br>2.27 (1.69 to 3.05)<br>Severe COVID-19: N=42 studies (12,056 patients)<br>2.21 (1.51-3.24)<br><br>CVD (CHD or Heart failure)<br>Mortality: N=51 studies (37,156 patients)<br>2.12 (1.77 to 2.56)<br>Severe COVID-19: N=73 studies (16,679 patients)<br>3.34 (2.71-4.1)<br><br>Cardiac arrhythmia<br>Mortality: N=6 studies (37,156 patients)<br>2.13 (1.72 to 2.65)<br>Severe COVID-19: N=4 studies (747 patients)<br>16.51 (6.69-40.77)<br><br>Arterial hypertension<br>Mortality: N=52 studies (31,341 patients)<br>2.02 (1.71 to 2.38)<br>Severe COVID-19: N=94 studies (20,817 patients) 2.5 (2.21- 2.92)<br><br>Diabetes mellitus

			Mortality: N=52 studies (31,341 patients)<br>1.84 (1.61 to 2.1)<br>Severe COVID-19: N=97 studies (21,381 patients)<br>2.51 (2.2-2.87)<br><br>Obesity<br>Mortality: N=3 studies (8,922 patients)<br>1.41 (1.15-1.74)<br>Severe COVID-19: N=8 studies (1,140 patients) 3.74 (2.37-5.89)<br><br>Dyslipidaemia<br>Mortality: N=4 studies (11,273 patients)<br>1.26 (1.06-1·5)<br>Severe COVID-19: N=4 studies (559 patients)<br>0.63 (0.22-1.83)
Jain, Yuan[28]<br><br>January 1, 2019, to March 5, 2020<br><br>N=7 studies (1,813 patients)<br>7 China<br><br>Case-control/cohort<br>STROBE Checklist:<br>1 Study <55% criteria met<br>4 studies 55-65% criteria met<br>2 studies >65% criteria met<br><br>AMSTAR2: Low quality	CVD<br>Hypertension<br>Diabetes mellitus	Severe COVID<br>ICU admission	OR (95% CI)<br><br>CVD<br>Severe COVID-19: N=3 studies (53 patients)<br>2.70 (1.52–4.80)<br>ICU: N=3 studies (75 patients)<br>4.44 (2.64–7.47)<br><br>Hypertension<br>Severe COVID-19: N=3 studies (212 patients)<br>1.97 (1.40–2.77)<br>ICU: N=3 studies (214 patients)<br>3.65 (2.22–5.99)<br><br>Diabetes mellitus<br>Severe COVID-19: N=3 studies (105 patients)<br>3.12 (1.00–9.75)<br>ICU: N=3 studies (103 patients)<br>2.72 (0.70–10.6)
Khan, Khan[29]<br><br>December 1, 2019 to April 31, 2020<br><br>N=41 studies (27,670 patients)<br>29 China, 4 Italy, 3 USA, 1 Australia, 1 Mexico, 1 Iran, 1 UK, 1 Korea<br><br>Case-control/cohort<br>Newcastle-Ottawa Scale, range 5-8<br><br>AMSTAR2: Low quality	CVD<br>Cerebrovascular disease<br>Renal disease<br>Liver diseases<br>Hypertension<br>Heart failure<br>Arrhythmia	Mortality	OR (95% CI)<br><br>Cerebrovascular disease<br>N=15 studies<br>4.12 (3.04-5.58) I^2=26%<br><br>Renal disease<br>N=21 studies<br>3.02 (2.60-3.51) I^2=56%<br><br>Liver diseases<br>N=13 studies<br>2.35 (1.50-3.69) I^2=0%<br><br>CVD<br>N=32 studies

			3.42 (2.86-4.09) I^2=84%<br><br>Hypertension<br>3.36 (2.64-4.28)<br><br>Heart failure<br>4.72 (3.19-6.97)<br><br>Arrhythmia<br>3.89 (2.51-6.02)
Kumar, Arora, Clinical Features[30]<br><br>January 1, 2020 and March 17, 2020<br><br>N=58 studies; 21 in meta-analyses (6,892 patients; 3,496 in meta-analyses)<br>53 China, 1 Hong Kong, 1 Singapore, 1 South Korea, 1 Australia, 1 Europe<br><br>Case-control/cohort<br>NIH tool, range 6-9 (50 good quality, 8 f fair quality, 0 poor quality).<br><br>AMSTAR2: Critically low quality	Comorbidity<br>Diabetes mellitus<br>Hypertension<br>CVD	Severe clinical course<br><br>(Patients in the primary studies with severe COVID-19, ICU, and/or mortality are labelled severe clinical course)	OR (95% CI)<br><br>Comorbidity<br>N=12 studies<br>3.16 (2.32-4.29) I^2=29%<br><br><br>Diabetes mellitus<br>N=14 studies<br>3.11 (1.99-4.88) I^2=48%<br><br>Hypertension<br>N=13 studies<br>2.30 (1.84-2.89) I^2= 3%<br><br>CVD<br>N=13 studies<br>3.88 (2.30-6.54) I^2=26%
Kumar, Arora, Diabetes[31]<br><br>January 01, 2020 to April 22, 2020<br><br>N=33 studies (16,003 patients)<br>30 China, 2 USA, 1 France<br><br>Case-control/cohort<br>NIH tool, range 7-9 (out of 12); 32 good quality, 1 study fair quality.<br><br>AMSTAR2: Low quality	Diabetes mellitus	Severe clinical course*<br>Severe COVID as labelled in primary studies<br>Mortality<br><br>*Patients in the primary studies with severe COVID-19, ICU, and/or mortality are labelled severe clinical course.	OR (95% CI)<br><br>Diabetes mellitus and severe clinical course<br>N=33 studies<br>2.49 (1.98-3.14) I^2=63%<br><br>Diabetes mellitus and severe COVID<br>N=24 studies<br>2.75 (2.09-3.62) I^2=63%<br><br>Diabetes mellitus and mortality<br>N=9 studies<br>1.90 (1.98-3.14) I^2=32%
Li, Guan[32]<br><br>January 01, 2020 to April 14, 2020<br><br>N=10 studies (3,118 patients)<br>10 China<br><br>Case-control/cohort, case-series	CVD<br>Acute cardiac injury	In-hospital mortality	OR (95% CI)<br><br>CVD<br>N=8 studies<br>4.85 (3.06-7.70) I^2=29%<br><br>Acute cardiac injury<br>N=8 studies<br>21.15 (10.19-43.94) I^2=71%

Newcastle-Ottawa scale, range 6-8<br><br>AMSTAR2: Low quality			
Li, He[33]<br><br>January 01, 2020 to April 14, 2020<br><br>N=12 studies (2,445 patients)<br>12 China<br><br>Case-control/cohort<br>Newcastle-Ottawa scale, range 6-8<br><br>AMSTAR2: Low quality	Smoking<br>Acute cardiac injury	Severe COVID<br>(ICU vs No ICU)	OR (95% CI)<br><br>Smoking history<br>Fixed effect<br>N=5 studies<br>1.70 (1.20-2.41) I^2=43%<br>Random effect<br>N=5 studies<br>1.62 (0.79-3.36) I^2=43%<br><br>Acute cardiac injury<br>Fixed effect<br>N=3 studies<br>3.38 (1.50-7.60) I^2=78%<br>Random effect<br>N=3 studies<br>4.35 (0.47-40.00) I^2=78%
Li, Huang[34]<br><br>January 01, 2020 to April 6, 2020<br><br>N=212 studies (281,461 patients)<br>180 China, 8 USA, 6 South Korea, 3 Singapore, 3 Italy, 3 Taiwan, 2 UK, 2 Hong Kong, 1 Canada, 1 Japan, 1 Vietnam, 2 multi-country<br><br>Case-control/cohort, case-series<br>Newcastle-Ottawa Scale, range 4-9 (average 7)<br><br>AMSTAR2: Low quality	Diabetes mellitus<br>Smoking<br>Cerebrovascular disease<br>CVD<br>Hypertension<br>Cardiac failure	Severe COVID*<br>Mortality<br><br>*Severe COVID-19 disease definition based on the WHO Interim Guidance Report or IDSA/ATS criteria for severe pneumonia	Meta-regression coefficient (95% CI)<br><br>Diabetes mellitus and severe COVID<br>23.4 (14.99-31.7) P<0.0001<br><br>Smoking severe COVID<br>-1.4 (9.7-6.9) P=0.7<br><br>Cerebrovascular disease severe COVID<br>19.6 (2.6-36.6) P=0.02<br><br>CVD and severe COVID<br>2.0 (3.4-7.4) P=0.5<br><br>Hypertension and severe COVID<br>5.1 (1.1-9.1) P=0.01<br><br>Cardiac failure and severe COVID<br>-37.2 (-81.2-6.7) P=0.1<br><br>Diabetes mellitus and mortality<br>8.2 (2.4-13.99) P=0.006<br><br>Smoking and mortality<br>-10.3 (29.7-9.2) P=0.3<br><br>Cerebrovascular disease and mortality<br>0.8 (6.0-7.7) P=0.8<br><br>Chronic heart disease and mortality<br>3.7 (0.96-8.4) P=0.1<br><br>Hypertension and mortality<br>6.99 (3.3-10.7) P=0.0002

			Cardiac failure and mortality<br>6.2 (2.3-10.1) P=0.002
Liu, Chen[35]<br><br>April 5, 2020<br><br>N=24 studies (10,948 patients)<br>20 China, 2 USA, 1 Italy, 1 France<br><br>Primary study design NR<br>Newcastle-Ottawa Scale, range 6-8<br><br>AMSTAR2: Low quality	Diabetes mellitus<br>Hypertension<br>CVD/CAD	Severe COVID*<br>ICU admittance<br>Mortality<br><br>*as defined in primary studies	OR (95% CI)<br><br>Comorbidity and Severe COVID<br>3.50 (1.78-6.90) I^2=61%<br><br>Comorbidity and ICU<br>3.36 (1.67-6.76) I^2=36%<br><br>Comorbidity and mortality<br>2.09 (0.26 to 16.67)<br><br>Diabetes mellitus and severe COVID<br>N=10 studies<br>2.61 (1.93-3.52) I^2=27%<br><br>Hypertension and severe COVID<br>N=9 studies<br>2.84 (2.22-3.63) I^2=37%<br><br>CVD and severe COVID<br>N=8 studies<br>4.18 (2.87-6.09) I^2=32%
Liu, Zhang[36]<br><br>Inception to April 13, 2020<br><br>N=36 studies (6,395 patients)<br>36 China<br><br>Case-control/cohort, case series<br>Newcastle-Ottawa scale, range 4-6 (31 studies =5, 4 studies=6, 1 study=4)<br><br>AMSTAR2: Critically low quality	Renal disease	Severe COVID	OR (95% CI)<br><br>Renal disease<br>3.28 (2.00-5.37) I^2=0%<br>N=13 studies (3,325 patients)
Lu, Zhong[37]<br><br>April 11, 2020<br><br>N=10 studies (11,818 patients)<br>7 China, 1 Italy, 1 Korea, 1 USA<br><br>Case-control/cohort, case-series<br>Newcastle-Ottawa Scale, range 5-9<br><br>AMSTAR2: Moderate quality	Comorbidity<br>Hypertension<br>Diabetes mellitus	Mortality	OR (95% CI)<br><br>Comorbidity<br>N=7 studies (2,517 patients)<br>3.50 (2.35-5.20) I^2=45%<br><br>Hypertension<br>N=6 studies (3,342 patients)<br>3.25 (2.15-4.91) I^2=69%<br><br>Diabetes mellitus<br>N=5 studies (2,307 patients)<br>2.63 (1.45-4.76) I^2=64%

Luo, Fu[38]<br><br>December 2019 to July 2020<br><br>N=124 studies<br>86 China, 10 USA, 7 Italy, 5 Korea, 2 Spain, 2 Switzerland, 2 Iran, 1 UK, 1 France, 1 Bolivia, 1 Egypt, 1 Greece, 1 Israel, 1 Netherlands, 1 Asia, EU, and USA, 1 Poland, 1 Japan<br><br>Primary study design NR<br>Newcastle-Ottawa scale, range 5-8 (out of 9)<br><br>AMSTAR2: Moderate quality	Hypertension<br>Diabetes mellitus<br>CVD<br>Renal disease<br>Acute cardiac injury	Severe COVID*<br>Mortality<br><br>*as defined in primary studies	OR (95% CI)<br><br>Hypertension<br>Severe COVID: N=55 studies<br>2.56 (2.12-3.11) I^2=83%<br>Mortality: N=58 studies<br>2.50 (2.02-3.11) I^2=93%<br><br>Diabetes mellitus<br>Severe COVID: N=58 studies<br>2.54 (1.89-3.41) I^2=89%<br>Mortality: N=63 studies<br>2.50 (2.02-3.11) I^2=93%<br><br>CVD<br>Severe COVID: N=29 studies<br>3.86 (2.70-5.52) I^2=63%<br>Mortality: N=30 studies<br>2.65 (1.86-3.78) I^2=86%<br><br>Renal disease<br>Severe COVID: N=28 studies<br>2.20 (1.26-3.85) I^2=77%<br>Mortality: N=35 studies<br>3.07 (2.43-3.88) I^2=73%<br><br>Acute cardiac injury<br>Severe COVID: N=11 studies<br>6.57 (3.70-11.65) I^2=75%<br>Mortality: N=14 studies<br>16.97 (7.87-36.57) I^2=89%
Ma, Gu[39]<br><br>Inception to February 25, 2020<br><br>N=30 studies (53,000 patients)<br>27 China, 1 USA, 1 Australia, 1 South Korea<br><br>Case-control/cohort<br>Agency for Healthcare Research and Quality, range 5-10<br><br>AMSTAR2: Low quality	Hypertension<br>Diabetes mellitus<br>CVD<br>Cerebrovascular disease<br>Renal disease	Severe COVID<br>Mortality	OR (95% CI)<br><br>Hypertension<br>Severe COVID: N=10 studies (2,511 patients)<br>2.06 (1.61-2.62) I^2=36%<br>Mortality:<br>4.48 (3.69-5.45)<br><br>Diabetes mellitus<br>Severe COVID: N=10 studies (2,511 patients)<br>2.49 (1.82-3.40) I^2=44%<br>Mortality:<br>4.43 (3.49-5.62)<br><br>CVD<br>Mortality:<br>6.75 (5.40-8.43)<br><br>Cerebrovascular disease<br>Severe COVID: N=5 studies (2,197 patients)<br>3.22 (1.49-6.97) I^2=0%<br>Mortality:<br>5.34 (2.34-12.16)

			Renal disease<br>Severe COVID: N=4 studies (1,620 patients)<br>6.02 (2.19-16.51) I^2=0%<br>Mortality:<br>9.02 (3.81-21.36)
Mantovani, Byrne[40]<br><br>January 1, 2020 to May 15, 2020<br><br>N=83 studies (78,874 patients)<br>62 Asia, 21 Europe, Australia, USA<br><br>Case-control/cohort<br>Newcastle-Ottawa scale, range 5-6<br><br>AMSTAR2: Moderate quality	Diabetes mellitus	Severe COVID*<br>In-hospital mortality<br><br>*as defined in primary studies	OR (95% CI)<br><br>Diabetes mellitus and severe COVID<br>N=22 studies<br>2.10 (1.71-2.57) I^2=42%<br><br>Diabetes mellitus and mortality<br>N=15 studies<br>2.68 (2.09-3.44) I^2=47%
Mao, Lin[41]<br><br>October 1, 2019 to July 26, 2020<br><br>N=17 studies (1,310 patients)<br>13 China, 1 England, 1 France, 1 South Korea, 1 Turkey<br><br>Case-control/cohort<br>Newcastle-Ottawa Study for cohort studies (12 moderate quality and 4 high quality) and AHRQ for cross-sectional studies (1 moderate quality)<br><br>AMSTAR2: Critically low quality	Amongst patients with COVID-19 and diabetes mellitus:<br><br>Hypertension<br>CVD<br>Cerebrovascular disease<br><br>For all patients:<br>Diabetes mellitus	Mortality<br>Severe COVID<br>Cardiac injury	OR (95% CI)<br><br>Patients with COVID-19 and diabetes mellitus:<br>Hypertension and mortality<br>N=3 studies (288 patients)<br>0.60 (0.12-3.11) I^2=47%<br><br>CVD and mortality<br>N=3 studies (288 patients)<br>0.44 (0.17-1.19) I^2=52%<br><br>Cerebrovascular disease and mortality<br>N=2 studies (201 patients)<br>0.32 (0.10-1.02) I^2=0%<br><br>All patients:<br>Diabetes mellitus and mortality<br>N=14 studies (3,699 patients)<br>2.52 (1.77-3.58) I^2=58%<br><br>Diabetes mellitus and severe COVID<br>N=9 studies (2,366 patients)<br>2.66 (2.12-3.32) I^2=33%<br><br>Diabetes mellitus and cardiac injury<br>N=7 studies (2,154 patients)<br>2.13 (1.66-2.73) I^2=47%
Matsushita, Ding[42]<br><br>December 1, 2019 to April 3, 2020	Smoking<br>Hypertension<br>Diabetes mellitus<br>CVD	Severe COVID (all-cause mortality, ICU admission, ARDS, or the need for mechanical ventilation)	OR (95% CI)<br><br>Current vs. never smoking<br>N=3 studies<br>1.82 (0.83-3.96) I^2=58%

N=25 studies (76,638 patients)<br>21 China, 3 USA, 1 Italy<br><br>Case-control/cohort, cross-sectional<br>Newcastle-Ottawa Scale, range 5-9<br><br>AMSTAR2: Moderate quality			Former vs. never smoking<br>N=3 studies<br>2.95 (1.15-7.53) I^2=65%<br><br>Hypertension<br>N=8 studies<br>3.08 (2.33-4.07) I^2=41%<br><br>Diabetes mellitus<br>N=9 studies<br>3.55 (2.56-4.93) I^2=61%<br><br>CVD<br>N=10 studies<br>5.05 (4.36-5.85) I^2=0%
Momtazmanesh, Shobeiri[43]<br><br>Inception to April 21, 2020<br><br>N=54 studies<br>Primary data origin NR<br><br>Case-control/cohort, case-series, case-reports<br>Newcastle-Ottawa scale, range 5-8<br><br>AMSTAR2: Moderate quality	Diabetes mellitus<br>Hypertension<br>CVD<br>Acute cardiac injury	Prevalence of new-onset CV conditions<br>- Acute cardiac injury<br>- Arrhythmia<br>- Heart failure<br><br>Mortality	Prevalence % (95% CI)<br><br>Acute cardiac injury<br>N=16 studies (2,647 patients)<br>25.3 (19.5-31.1) I^2=93%<br><br>Arrhythmia<br>N=4 studies (444 patients)<br>26.1 (5.9-46.1) I^2=97%<br><br>Heart failure<br>N=2 studies (367 patients)<br>23.7 (19.3-28.0) I^2=0%<br><br>OR (95% CI)<br><br>Acute cardiac injury and mortality<br>N=7 studies (1,046 patients)<br>19.64 (10.28-37.53) I^2=64%<br><br>CVD and mortality<br>N=6 studies (550 patients)<br>7.87 (2.117-28.57) I^2=54%<br><br>Hypertension and mortality<br>N=8 studies (1,033 patients)<br>2.49 (2.02-3.07) I^2=25%<br><br>Diabetes mellitus and mortality<br>N=6 studies (682 patients)<br>1.66 (1.20-2.29) I^2=0%
Moula, Micali[44]<br><br>December 1, 2019 to May 18, 2020<br><br>N=26 studies (8,497 patients)<br>20 China, 2 Italy, 2 Korea, 1 Iran, 1 USA<br><br>Case-control/cohort, case-series	CVD and CAD<br>Hypertension<br>Cerebrovascular disease<br>Diabetes mellitus	Mortality	RR (95% CI)<br><br>CVD/CAD<br>N=26 studies<br>1.96 (1.51-2.54)<br><br>Hypertension<br>N=24 studies<br>1.73 (1.37-2.19)

ROBINS-I tool, overall bias ratings were: 10 Critical, 6 Serious, 11 Moderate<br>(one paper split into two cohorts = '27 studies')<br><br>AMSTAR2: Low quality			Cerebrovascular disease<br>N=15 studies<br>1.76 (1.25-2.50)<br><br>Diabetes mellitus<br>N=26 studies<br>1.59 (1.25-2.02)
Nannoni, de Groot[45]<br><br>December 1, 2019 to September 14, 2020<br><br>N=145 studies (108,571 patients)<br>12 North America, 6 EU, 6 Asia<br><br>Case-control/cohort, case-series, case-reports<br>Newcastle-Ottawa Scale, 14 High quality, 19 Moderate quality.<br><br>AMSTAR2: Critically low quality	Severe COVID-19<br>Hypertension<br>Diabetes mellitus<br>CAD	Acute cerebrovascular disease	Proportion % (95% CI)<br><br>Acute cerebrovascular disease<br>N=24 studies (108,571 patients)<br>1.4 (1.0–1.9) I^2=95%<br><br>OR (95% CI)<br><br>Hypertension and acute cerebrovascular disease<br>N=4 studies (11,683 patients)<br>7.35 (1.94-27.87) I^2=76%<br><br>Diabetes mellitus and acute cerebrovascular disease<br>N=4 studies (11,683 patients)<br>5.56 (3.34-9.24) I^2=22%<br><br>CAD and acute cerebrovascular disease<br>N=2 studies (2,181 patients)<br>3.12 (1.61-6.02) I^2=0%<br><br>Severe COVID and acute cerebrovascular disease<br>N=3 (2,389)<br>5.10 (2.72-9.54) I^2=0%
Nasiri, Haddadi[46]<br><br>January 1, 2019 to May 29, 2020<br><br>N=34 studies (5,057 patients)<br>32 China, 1 Germany, 1 Norway<br><br>Case-control/cohort, case-series, case-reports, cross-sectional<br>Joanna Briggs Institute (JBI) checklist, all classified as low risk of bias.<br><br>AMSTAR2: Moderate quality	COVID-19	Acute cardiac injury	Pooled frequency (95%CI)<br><br>Acute cardiac injury<br>N=243 patients<br>12.4 (6.2-23.2) I^2=65%
Noor, Islam[47]<br><br>January 1, 2020 to August 11, 2020	Obesity<br>Smoking<br>Hypertension<br>Diabetes mellitus<br>CVD<br>Cerebrovascular	Mortality	RR (95% CI)<br><br>Obesity<br>N=7 studies (13,477 patients)<br>2.18 (1.10–4.34) I^2=99%

N=58 studies (122,191 patients)<br>26 China, 8 USA, 7 Italy, 4 Spain, 2 South Korea, 2 Mexico, 1 Bangladesh, 1 Brazil, 1 UK, 1 Greece, 1 Iran, 1 Kuwait, 1 Switzerland, 1 Turkey, 1 EU<br><br>Case-control/cohort<br>Newcastle-Ottawa scale, range 6-9<br><br>AMSTAR2: Moderate quality	disease<br>CHD<br>Renal disease<br>Liver disease		Smoking<br>N=10 studies (13,598 patients)<br>1.81 (0.99–3.33) I^2=99%<br><br>Hypertension<br>N=38 studies (37,785 patients)<br>2.08 (1.79–2.43) I^2=98%<br><br>Diabetes mellitus<br>N=35 studies (35,411 patients)<br>1.87 (1.23–2.84) I^2=100%<br><br>CVD<br>N=16 studies (8,925 patients)<br>2.51 (1.20–5.26) I^2=100%<br><br>Cerebrovascular disease<br>N=11 studies (6,069 patients)<br>2.75 (1.54–4.89) I^2=99%<br><br>CHD<br>N=11 studies (10,851 patients)<br>3.63 (1.52–8.65) I^2=100%<br><br>Renal disease<br>N=16 studies (24,450 patients)<br>2.11 (1.72–2.58) I^2=97%<br><br>Liver disease<br>N=8 studies (7,090 patients)<br>2.02 (1.16–3.50) I^2=95%
Palaiodimos, Chamorro-Pareja[48]<br><br>May 10, 2020<br><br>N=14 studies (18,506 patients)<br>5 Asia, 5 USA, 4 EU<br><br>Case-control/cohort<br>Quality in Prognosis Studies (QUIPS) tool, all low risk of bias<br><br>AMSTAR2: Moderate quality	Diabetes mellitus	Mortality	OR (95% CI)<br><br>Diabetes mellitus<br>N=14 studies (18,506 patients)<br>1.65 (1.35-1.96) I^2=77%
Parohan, Yaghoubi[49]<br><br>Inception to May 1, 2020<br><br>N=14 studies (29,909 patients)<br>12 China, 1 Italy, 1 Iran<br><br>Case-control/cohort<br>Newcastle-Ottawa scale, range 5-8	Hypertension<br>CVD<br>Diabetes mellitus	Mortality	OR (95% CI)<br><br>Hypertension<br>N=8 studies<br>2.70 (1.40-5.24) I^2=93%<br><br>CVD<br>N=9 studies<br>3.72 (1.77-7.83) I^2=89%

AMSTAR2: Critically low quality			Diabetes mellitus<br>N=7 studies<br>2.41 (1.05-5.51) I^2=94%
Parveen, Sehar[50]<br><br>Inception to March 31, 2020<br><br>N=7 studies (2,018 patients)<br>7 China<br><br>Case-control/cohort, case-series<br>NIH Quality Assessment tool, 4 Good, 3 Fair<br><br>AMSTAR2: Critically low quality	Diabetes mellitus<br>Hypertension	Prevalence in of diabetes mellitus/hypertension	OR (95% CI)<br><br>Diabetes mellitus in non-survivors vs. survivors<br>N=2 studies<br>0.56 (0.35-0.90) I^2=0%<br><br>Diabetes mellitus in ICU vs non-ICU<br>N=2 studies<br>0.78 (0.06-9.34) I^2=76%<br><br>Hypertension in non-survivors vs survivors<br>N=2 studies<br>0.50 (0.34-0.73) I^2=0%<br><br>Hypertension in ICU vs non-ICU<br>N=2 studies<br>0.42 (0.22-0.81) I^2=0%
Patanavanich, Glantz[51]<br><br>January 1, 2020 to May 25, 2020<br><br>N=47 studies (31,871 patients)<br>33 China, 8 USA, 3 Italy, 1 UK, 1 South Korea, 1 International<br><br>Case-control/cohort, case-series<br>Modified ACROBAT-NRSI tool, range 0-1.6<br><br>AMSTAR2: Moderate quality	Smoking	Severe COVID*<br>Mortality<br><br>*Respiratory distress with respiratory rate ≥30/min, or oxygen saturation ≤93% at rest, or oxygenation index ≤300 mmhg.	OR (95% CI)<br><br>Smoking and severe COVID<br>N=47 studies<br>1.56 (1.32-1.83) I^2=45%<br><br>Smoking and mortality<br>N=8 studies<br>1.19 (1.05-1.34) I^2=0%
Patel, Malik, Shah[52]<br><br>December 1, 2019 to April 30, 2020<br><br>N=11 studies (4,987 patients)<br>Primary study origin NR<br><br>Case-control/cohort<br>Newcastle-Ottawa Scale, range 4-6<br>Cochrane's Collaboration Tool (3 high risk of bias, 8 moderate risk of bias)<br><br>AMSTAR2: Critically low quality	Cerebrovascular disease	ICU admission<br>Mechanical ventilation<br>Mortality<br>Composite outcome	OR (95% CI)<br><br>ICU admission unadjusted<br>N=7 studies (3,901 patients)<br>1.54 (1.25-1.62) I^2=95%<br><br>ICU admission age-adjusted<br>N=7 studies (3,901 patients)<br>1.82 (1.25-2.69) I^2=94%<br><br>Mechanical ventilation unadjusted<br>N=8 studies (2,196 patients)<br>1.32 (1.13-1.55) I^2=91%<br><br>Mechanical ventilation age-adjusted<br>N=8 studies (2,196 patients)

			1.33 (1.09-1.63) I^2=93%<br><br>Mortality unadjusted<br>N=8 studies (4,240 patients)<br>1.45 (1.22-1.72) I^2=96%<br><br>Mortality age-adjusted<br>N=8 studies (4,240 patients)<br>1.42 (1.14-1.77) I^2=96%<br><br>Composite outcome<br>2.67 (1.75-4.06) I^2=12%
Patel, Malik, Usman[53]<br><br>December 1, 2019 to May 31, 2020<br><br>N=29 studies (12,258 patients)<br>19 China, 5 USA, 2 Singapore, 1 Australia, 1 Europe, 1 South Korea<br><br>Case-control/cohort<br>Newcastle-Ottawa Scale, 12 high risk of bias, 17 moderate risk of bias<br><br>AMSTAR2: Critically low quality	Smoking<br>Diabetes mellitus<br>Hypertension<br>Cerebrovascular disease<br>Chronic liver disease<br>CVD<br>Cardiac complications	Mortality<br>Mechanical ventilation	OR (95% CI)<br><br>Smoking<br>Mechanical ventilation<br>0.9 (0.88-0.97) I^2=94%<br>Mortality<br>0.95 (0.93-0.98) I^2=81%<br><br>Diabetes mellitus<br>Mechanical ventilation<br>1.02 (0.94-1.11) I^2=96%<br>Mortality<br>1.02 (0.93-1.12) I^2=96%<br><br>Hypertension<br>Mechanical ventilation<br>1 (0.94-1.11) I^2=96%<br>Mortality<br>1.01 (0.93-1.09) I^2=96%<br><br>Cerebrovascular disease<br>Mechanical ventilation<br>1.42 (1.14-1.77) I^2=96%<br>Mortality<br>1.34 (1.09-1.63) I^2=93%<br><br>Chronic liver disease<br>Mechanical ventilation<br>1.08 (1.01-1.17) I^2=96%<br>Mortality<br>1.08 (1.03-1.17) I^2=94%<br><br>CVD<br>Mechanical ventilation<br>0.99 (0.88-1.12) I^2=96%<br>Mortality<br>1.32 (1.1-1.58) I^2=90%<br><br>Cardiac complications<br>Mechanical ventilation<br>1.01 (0.92-1.11) I^2=95%<br>Mortality<br>0.98 (0.9-1.06) I^2=95%
Porto, Iamonti[54]	Diabetes mellitus	Mortality	OR (95% CI)

Inception to April 2020<br><br>N=5 studies (1,453 patients)<br>5 China<br><br>Primary study design NR<br>Jedad scale, only studies scoring ≥2 points were included and considered high quality<br><br>AMSTAR2: Critically low quality			Diabetes mellitus<br>8.9 (4.5-17.4)
Reddy, Charles[55]<br><br>December 1, 2019 to June 2, 2020<br><br>N=47 studies (32,849 patients)<br>32 China, 10 USA, 2 International, 1 UK, 2 Italy<br><br>Case-control/cohort<br>Newcastle-Ottawa scale, 22 Good quality, 6 Fair quality, 19 Poor quality<br><br>AMSTAR2: High quality	Current smoking vs. Former/never<br>Smoking history vs. Never	Severe or critical COVID<br>Mortality<br>Disease progression<br>ICU admission<br>Mechanical ventilation	RR (95% CI)<br><br>Current smoking<br>Severe/critical COVID: N=8 studies (2,100 patients)<br>1.98 (1.16-3.38) I^2=87%<br>Mortality: N=7 studies (14,741)<br>1.46 (0.83-2.60) I^2=81%<br>N=7 (14,741 patients)<br>Disease progression: N=3 studies (458 patients)<br>1.54 (0.52-4.58) I^2=81%<br>ICU admission: N=6 studies (2,368 patients)<br>0.72 (0.42-1.24) I^2=40%<br>Mechanical ventilation: N=5 studies (1,585 patients)<br>1.13 (0.75-1.72) I^2=32%<br><br>Smoking history<br>Severe/critical COVID: N=15 studies (4,007 patients)<br>1.35 (1.19-1.53) I^2=19%<br>Mortality: N=9 studies (14,105 patients)<br>1.26 (1.20-1.32) I^2=0%<br>Disease progression: N=5 studies (468 patients)<br>2.18 (1.06-4.49) I^2=69%<br>ICU admission: N=4 studies (1,802 patients)<br>1.12 (0.96-1.31) I^2=0%<br>Mechanical ventilation: N=4 studies (917 patients)<br>1.20 (1.01-1.42) I^2=0%
Rhim, Park[56]<br><br>Inception to May 1, 2020<br><br>N=23 studies (227,856 patients)<br>19 China, 1 Italy, 1 Spain, 1 USA, 1 Korea	Diabetes mellitus<br>Hypertension<br>CVD<br>Cerebrovascular disease<br>Chronic liver disease<br>Renal disease<br>Acute cardiac injury	Mortality	OR (95% CI)<br><br>Diabetes mellitus<br>2.53 (1.77-3.61) I^2=66%<br>N=15 studies (206,488 patients)<br><br>Hypertension<br>2.88 (2.22-3.73) I^2=7%<br>N=11 studies (1,246 patients)

Case-control/cohort, cross-sectional, case series<br>Newcastle-Ottawa Scale, range 7-9<br><br>AMSTAR2: Low quality			CVD<br>5.06 (3.54-7.24) I^2=46%<br>N=14 studies (206,287 patients)<br><br>Cerebrovascular disease<br>4.83 (2.61-8.93) I^2=34%<br>N=9 studies (1,306 patients)<br><br>Chronic liver disease<br>1.36 (0.68-2.74) I^2=1%<br>N=6 studies (812 patients)<br><br>Renal disease<br>6.32 (3.62-11.03) I^2=0%<br>N=8 studies (1,500 patients)<br><br>Acute cardiac injury<br>Cases: 43%<br>22.7 (4.81-107.2) I^2=81%<br>N=4 studies (582 patients)
Roncon, Zuin[57]<br><br>Inception to March 25, 2020<br><br>N=8 studies (1,382 patients)<br>Primary study origin NR<br><br>Primary study design NR<br>Newcastle-Ottawa Scale, 7 high quality (>7 stars), 1 moderate quality (5-7 stars)<br><br>AMSTAR2: Critically low quality	Diabetes mellitus	ICU admission<br>Mortality	OR (95% CI)<br><br>Diabetes mellitus and ICU admission<br>N=4 studies (114 patients)<br>2.79 (1.85-4.22), I^2=46%<br><br>Diabetes mellitus and mortality<br>N=4 (354 patients)<br>3.21 (1.82, 5.64) I^2=16%
Sabatino, De Rosa[58]<br><br>December 1, 2019 to June 11, 2020<br><br>N=21 studies (77,317 patients)<br><br>11 China, 5 USA, 1 Italy, 1 UK, 1 Singapore, 1 Korea, 1 Iran<br><br>Primary study design NR<br>Quality Assessment Tool for Observational<br>Cohort and Cross-Sectional Studies (QAT-OC/CSS) of the NIH, overall quality rating was<br>judged as Fair<br><br>AMSTAR2: Moderate quality	COVID-19<br><br><br><br><br><br><br><br>Cardiovascular co-morbidity/risk factor<br>Cardiovascular complications	Angina<br>Arrhythmias<br>Myocardial injury<br>Acute Heart failure<br>Myocardial infarction<br>CV complications<br><br><br><br>Mortality	Prevalence % (95% CI)<br><br>Angina<br>10.2 (3.2-20.5)<br><br>Arrhythmias<br>18.4 (7.8-32.3)<br><br>Myocardial injury<br>10.3 (6.7-14.6)<br><br>Acute Heart failure<br>2.0 (0.94-3.35)<br><br>Myocardial infarction<br>3.5 (2.1-5.3)<br><br>Cardiovascular complications<br>14.1 (10.3-20.2)<br><br>Associations with mortality, regression coefficient 95%CI

			Cardiovascular comorbidity<br>0.004 (0.003-0.005) p<0.001<br><br>Cardiovascular complications<br>0.001 (0.000-0.003) p=0.038
Sales-Peres, Azevedo-Silva[59]<br><br>Inception to April 27, 2020<br><br>N=9 studies (6,577 patients)<br>3 USA, 2 China, 2 France, 1 Spain, 1 Italy<br><br>Case-control/cohort, cross-sectional, case series<br>Newcastle-Ottawa Scale, range 6-8<br><br>AMSTAR2: Critically low quality	Obesity	Severe complications	RR (95% CI)<br><br>Obesity<br>N=3 studies (463 patients)<br>1.40 (0.91-2.17) I^2=38%
Sepandi, Taghdir[60]<br><br>January 1, 2020 to March 23, 2020<br><br>N=13 studies (12,044 patients)<br><br>13 China<br>Case-control/cohort<br>Newcastle-Ottawa Scale, 2 Fair, 11 Good<br><br>AMSTAR2: Critically low quality	Diabetes mellitus<br>Hypertension<br>Renal disease<br>CVD<br>Smoking	Mortality	OR (95% CI)<br><br>Diabetes mellitus<br>N=9 studies<br>2.42 (1.06-5.52) I^2=90%<br><br>Hypertension<br>N=8 studies<br>2.54 (1.21-5.32) I^2=91%<br><br>Renal disease<br>N=7 studies<br>2.61 (1.22-5.60) I^2=78%<br><br>CVD<br>N=6 studies<br>4.37 (1.13-16.9) I^2=88%<br><br>Smoking<br>N=3 studies<br>1.70 (0.53-5.35) I^2=43%
Shafi, Shaikh[61]<br><br>Search dates NR<br><br>N=61 studies<br>33 China, 10 USA, 5 Italy, 3 Spain, 2 Germany, 1 International, 1 France and Switzerland, 1 South Korea, 1 France, 1 Belgium, 1 Iran, 1 Trinidad, 1 Brazil<br><br>Case-control/cohort, case series, case report, RCT	COVID-19	Cardiovascular and cardiac manifestations	Patients with hypertension or any other cardiovascular comorbidity were more likely to develop a cardiovascular complication due to SARS-CoV-2 infection, with a higher proportion of hypertensive patients developing acute heart injury and heart failure.<br><br>Patients affected with COVID-19 are at an increased risk of arrhythmias due to underlying comorbidities, polypharmacy, and disease progression.

Newcastle-Ottawa Scale, range 5-9<br><br>AMSTAR2: Low quality			Myocardial injury in COVID-19 is a recognized phenomenon. Case series include reports of myocarditis, ACS, and spontaneous coronary artery dissection. Cardiac biomarkers are important in recognizing patients that might be presenting with early signs of myocardial injury secondary to COVID-19.
Shao, Shang[62]<br><br>Inception to March 31, 2020<br><br>N=9 studies (1,470 patients)<br>9 China<br><br>Primary study design NR<br>Newcastle-Ottawa Scale, range 7-8 (7 studies scored 8 and 2 studies scored 7)<br><br>AMSTAR2: Low quality	Myocardial injury	Mortality	OR (95% CI)<br><br>Myocardial injury<br>13.7 (9.8-19.1) I^2=52%
Shi, Wang[63]<br><br>December 1, 2019 to April 29, 2020<br><br>N=27 studies<br>24 China, 2 USA, 1 Italy<br><br>Case-control/cohort<br><br>Quality in Prognostic Factor Studies (QUIPS) tool, range low risk in all categories-high risk in 3 categories<br><br>AMSTAR2: Low quality	Current smoking<br>Renal disease<br>Cerebrovascular disease<br>CVD<br>Diabetes mellitus<br>Hypertension<br>Chronic liver disease<br>Acute cardiac injury	Mortality	RR (95% CI)<br><br>Current smoking<br>N=5 studies (2,761 patients)<br>2.95 (1.32-6.58) I^2=30%<br><br>Renal disease<br>N=4 studies (2,111 patients)<br>8.37 (3.94-17.77) I^2=0%<br><br>Cerebrovascular disease<br>N=4 studies (2,071 patients)<br>7.66 (3.87-15.2) I^2=0%<br><br>CVD<br>N=5 studies (2,258 patients)<br>3.16 (2.19-4.56) I^2=17%<br><br>Diabetes mellitus<br>N=5 studies (2,689 patients)<br>2.21 (1.37-3.56) I^2=45%<br><br>Hypertension<br>N=6 studies (2,880 patients)<br>2.11 (1.49-2.99) I^2=82%<br><br>Chronic liver disease<br>N=3 studies (2,109 patients)<br>1.47 (0.63-3.42) I^2=0%<br><br>Acute cardiac injury<br>N=6 studies (1,207 patients)<br>8.22 (4.95-13.7) I^2=72%
Sinclair, Zhu[64]	COVID-19	Cardiac complications	Cardiac complications - prevalence

December 1, 2019 to May 11, 2020<br><br>N=5 studies (1,053 patients)<br>4 China, 1 USA<br><br>Primary study design NR<br>Newcastle Ottawa Scale, all 7 (high quality)<br><br>AMSTAR2: Moderate quality	CVD<br>Hypertension<br>Diabetes mellitus		17%<br><br>OR (95% CI) – cardiac complications<br><br>CVD<br>Fixed-effect: 5.12 (3.09-8.48)<br>Random-effect: 3.82 (1.44-10.15)<br>I^2=33%<br><br>Hypertension<br>Fixed-effect: 4.37 (2.99-6.39)<br>Random-effect: 4.35 (2.96-6.38) I^2=0%<br><br>Diabetes mellitus<br>Fixed-effects: 2.61 (1.67-4.09)<br>Random-effects: 2.40 (1.51-3.82) I^2=0%
Sreenivasan, Khan[65]<br><br>November 30, 2019 to March 30, 2019<br><br>N=10 studies (1,427 patients)<br>8 China, 1 Singapore, 1 USA<br><br>Case-control/cohort<br>Newcastle-Ottawa Scale, range 3-7<br><br>AMSTAR2: Critically low quality	COVID-19<br><br><br><br><br><br><br>Renal disease<br>Chronic liver disease<br>Smoking<br>CVD<br>Cerebrovascular disease<br>Diabetes mellitus<br>Hypertension	Acute myocardial infarction/injury<br>Arrhythmia<br><br><br><br><br>Complicated hospital course*<br><br>*Mortality, ICU admission, acute respiratory distress syndrome, or need for invasive mechanical ventilation.	Event rate (95% CI)<br><br>Acute myocardial infarction/injury<br>0.079 (0.029-0.197, p<0.001)<br>N=441 patients<br><br>Arrhythmia<br>0.167 (0.113-0.238, p<0.001)<br>N=138 patients<br><br>OR (95% CI) – Complicated hospital course<br><br>Renal disease<br>N=3 studies<br>5.12 (1.18-22.19) I^2=0%<br><br>Chronic liver disease<br>N=2 studies<br>1.07 (0.30-3.87)<br><br>Smoking<br>N=2 studies<br>2.54 (1.00-6.46)<br><br>CVD<br>N=6 studies<br>5.82 (2.44-13.85) I^2=28%<br><br>Cerebrovascular disease<br>N=2 studies<br>8.30 (1.24-55.4) I^2=0%<br><br>Diabetes mellitus<br>N=6 studies<br>2.46 (1.37-4.42) I^2=23%<br><br>Hypertension<br>N=6 studies<br>2.09 (1.02-4.27) I^2=74%

Ssentongo, Ssentongo[66]<br><br>December 1, 2019 to July 9, 2020<br><br>N=25 studies (65,484 patients)<br>19 China, 3 USA, 1 Italy, 1 Africa, 1 International<br><br>Case-control/cohort, case series<br>Newcastle-Ottawa Scale, range 5-9 (mean 7)<br><br>AMSTAR2: Moderate quality	CVD<br>Hypertension<br>Cerebral vascular disease<br>Diabetes mellitus<br>Renal disease<br>Chronic liver disease<br>Heart failure	Mortality	RR (95% CI)<br><br>CVD<br>N=14 studies<br>2.25 (1.60-3.17) I^2=49%<br><br>Hypertension<br>N=13 studies<br>1.82 (1.43-2.32) I^2=70%<br><br>Cerebral vascular disease<br>N=4 studies<br>2.16 (0.97-4.80) I^2=64%<br><br>Diabetes mellitus<br>N=16 studies<br>1.48 (1.02-2.15) I^2=84%<br><br>Renal disease<br>N=9 studies<br>3.25 (1.13-9.28) I^2=99%<br><br>Chronic liver disease<br>N=3 studies<br>1.73 (0.86-3.46) I^2=0%<br><br>Heart failure<br>N=3 studies<br>2.03 (1.28-3.21) I^2=0%
Tabrizi, Lankarani[67]<br><br>Inception to March 12, 2020<br><br>N=17 studies (3,189 patients)<br>16 China, 1 Singapore<br><br>Case-control/cohort<br>Newcastle Ottawa Scale, range 3-8<br><br>AMSTAR2: Critically low quality	Diabetes mellitus<br>Hypertension<br>CVD<br>Renal disease<br>Cerebrovascular accident<br>Liver disease	Severe COVID<br>(as measured by disease severity criteria as severe/critical disease type or admitted to ICU or the use of mechanical ventilation)	OR (95% CI)<br><br>Diabetes mellitus<br>N=9 studies<br>3.54 (1.79-7.01) I^2=58%<br><br><br>Hypertension<br>N=10 studies<br>2.35 (1.83-3.02) I^2=0%<br><br>CVD<br>N=9 studies<br>2.44 (1.64-3.63) I^2=0%<br><br>Renal disease<br>N=6 studies<br>6.38 (3.23-12.59) I^2=0%<br><br>Cerebrovascular accident<br>N=4 studies<br>3.94 (0.88-17.59) I_2=53%<br><br>Liver disease<br>N=6 studies<br>1.25 (0.35-4.41) I^2=40%
Tamara, Tahapary[68]	Obesity (BMI >25 or 30 kg/m2)	In-hospital critical care	One study demonstrated that COVID-19 patients with obesity grade II had 7.36

Inception to April 14, 2020<br><br>N=3 studies (806 patients)<br>1 China, 1 USA, 1 France<br><br>Case-control/cohort<br>Newcastle Ottawa Scale, range 7-9<br><br>AMSTAR2: Moderate quality			(1.63-33.14; p= 0.021) times increased risk of having invasive mechanical ventilation during in-hospital care, compared to non-obese patients with COVID-19.<br><br>One study stratified patients by age, <60 years and >60 years. Compared to healthy weight and over-weight groups, the rate of hospitalization increased by 2.0 (1.6-2.6; p<0.0001) and 2.2 (1.7-2.9; p< 0.0001) times in the younger patient group with obesity grade I and II, respectively.<br><br>Another study reported an increased risk of 1.30 (1.09-1.54; p<0.003) times in COVID-19 patients with a BMI higher than 25 kg/m^2 to develop severe COVID-19 compared to healthy weight and over-weight patients, however, this was attenuated in multivariate analyses.
Taylor, Hofmeyr[69]<br><br>January 1, 2020 to April 7, 2020<br><br>N=9 studies (1,823 patients)<br>7 China, 1 USA, 1 Italy<br><br>Case-control/cohort<br>Newcastle-Ottawa Scale, range 4-6<br><br>AMSTAR2: Critically low quality	Hypertension	Mortality in intensive care	OR (95% CI)<br><br>Hypertension<br>N=3 studies<br>4.17 (2.90-5.99) I^2=0%
Tian, Jiang[70]<br><br>January 1, 2020 to April 24, 2020<br><br>N=14 studies (4,659 patients)<br>13 China, 1 USA<br><br>Primary study design NR<br>Agency for Healthcare Research<br>and Quality (AHRQ) score checklist, 1 low quality, 5 moderate quality, 8 high quality<br><br>AMSTAR2: Critically low quality	Hypertension<br>CVD<br>Cerebrovascular disease<br>Diabetes mellitus<br>Smoking<br>Renal disease	Mortality	OR (95% CI)<br><br>Hypertension<br>N=11 studies (4,263 patients)<br>2.53 (2.07-3.09) I^2=15%<br><br>CVD<br>N=12 studies (1,842 patients)<br>3.81 (2.11-6.85) I^2=61%<br><br>Cerebrovascular disease<br>N=6 studies (948 patients)<br>4.92 (1.54-15.68) I^2=51%<br><br>Diabetes mellitus<br>N=12 studies (4,315 patients)<br>1.97 (1.67-2.31) I^2=0%<br><br>Smoking<br>N=4 studies (678 patients)<br>1.77 (0.83-3.81) I^2=8%

			Renal disease<br>N=6 studies (1,087 patients)<br>9.41 (3.23-27.40) I^2=0%
Villalobos, Ott[71]<br><br>Inception to April 29, 2020<br><br>N=75 studies<br>66 China, 5 USA, 2 UK, 1 Iran, 1 France<br><br>Primary study design NR<br>ROBINS-I tool<br>45 high risk of bias, only few had overall low risk of bias in all categories.<br><br>AMSTAR2: Moderate quality	CVD<br>Cerebrovascular disease<br>Renal disease<br>Diabetes mellitus<br>Liver disease<br>Hypertension<br>Myocardial infarction	ICU admission<br>Mortality	RR (95% CI)<br><br>CVD<br>ICU: N=8 studies<br>2.1 (1.3-3.2) I^2=86%<br>Mortality: N=15 studies<br>3.3 (2.3-4.5) I^2=86%<br><br>Cerebrovascular disease<br>ICU: N=4 studies<br>1.9 (0.9-4.0) I^2=92%<br>Mortality: N=7 studies<br>2.6 (1.7-4.1) I^2=61%<br><br>Renal disease<br>ICU: N=4 studies<br>2.1 (0.9-4.9) I^2=90%<br>Mortality: N=3 studies<br>2.5 (1.8-3.4) I^2=0%<br><br>Diabetes mellitus<br>ICU: N=12 studies<br>1.9 (1.4-2.6) I^2=90%<br>Mortality: N=18 studies<br>2.2 (1.7-2.9) I^2=83%<br><br>Hypertension<br>ICU: N=9 studies<br>1.4 (1.1-1.7) I^2=53%<br>Mortality: N=17 studies<br>2.7 (2.1-3.4) I^2=80%<br><br>Liver disease<br>Mortality: N=3 studies<br>1.9 (0.6-6.4) I^2=30%<br><br>Myocardial infarction<br>Mortality: N=5 studies<br>3.9 (1.5-8.6) I^2=89%
Wang, Deng[72]<br><br>December, 2019 to March 16, 2020<br><br>N=25 studies (4,881 patients)<br>25 China<br><br>Case-control/cohort, cross-sectional<br>Newcastle-Ottawa scale, range 3-7	Diabetes mellitus<br>Hypertension<br>CVD<br>Chronic liver disease	Severe COVID	RR (95% CI)<br><br>Diabetes mellitus<br>N=12 studies (1,740 patients)<br>1.53 (1.29-1.82)<br><br>Hypertension<br>N=13 studies (1,781 patients)<br>1.40 (1.22-1.60)<br><br>CVD<br>N=12 studies (1,412 patients)<br>1.79 (1.50-2.13)

AMSTAR2: Critically low quality			Chronic liver disease<br>N=8 studies (1,312 patients)<br>0.93 (0.62-1.42)
Wang, Li[73]<br><br>Inception to March 1, 2020<br><br>N=6 studies (1,558 patients)<br>6 China<br><br>Case-control/cohort<br>Newcastle-Ottawa Scale, range 6-8<br><br>AMSTAR2: Critically low quality	Hypertension<br>Diabetes<br>Liver disease<br>Renal disease<br>CVD<br>Cerebrovascular disease	Severe COVID	OR (95% CI)<br><br>Hypertension<br>N=6 studies<br>2.29 (1.69-3.10) I^2=4%<br><br>Diabetes<br>N=6 studies<br>2.47 (1.67-3.66) I^2=39%<br><br>Liver disease<br>N=5 studies<br>0.67 (0.30-1.49) I^2=0%<br><br>Renal disease<br>N=4 studies<br>2.51 (0.93-6.78) I^2=0%<br><br>CVD<br>N=4 studies<br>2.93 (1.73-4.96) I^2=0%<br><br>Cerebrovascular disease<br>N=3 studies<br>3.89 (1.64-9.22) I^2=45%
Wu, Liu[74]<br><br>Inception to April 1, 2020<br><br>N=41 studies (5,064 patients)<br>41 China<br><br>Case-control/cohort<br>Newcastle-Ottawa Scale, range 7-8 (2 studies =7, 39 studies =8)<br><br>AMSTAR2: Moderate quality	Smoking<br>Alcohol<br>Diabetes mellitus<br>CVD<br>Cerebrovascular disease<br>Hypertension<br>Chronic liver disease<br>BMI	Severe COVID	OR (95% CI)<br><br>Smoking<br>N=7 studies (1,484 patients)<br>1.26 (0.69-2.32)<br><br>Diabetes mellitus<br>N=17 studies (2,476 patients)<br>2.38 (1.59-3.57)<br><br>CVD<br>N=13 studies (2,089 patients)<br>3.16 (2.19-4.56)<br><br>Cerebrovascular disease<br>N=7 studies (1,213 patients)<br>3.34 (1.29-8.69)<br><br>Hypertension<br>N=18 studies (2,510 patients)<br>2.63 (1.79-3.88)<br><br>Chronic liver disease<br>N=11 studies (1,982 patients)<br>0.81 (0.47-1.40)<br><br>SMD (95% CI)<br><br>BMI

			N=4 studies (221 patients)<br>1.27 (-0.88-3.42)
Wu, Tang[75]<br><br>Inception to April 14, 2020<br><br>N=9 studies (926 patients)<br>9 China<br><br>Primary study origin NR<br>Newcastle-Ottawa scale, range 7-8 (9 studies=7 and 1 study =8)<br><br>AMSTAR2: Moderate quality	Diabetes mellitus	Mortality	OR (95% CI)<br><br>Diabetes mellitus<br>1.75 (1.31-2.36) I^2=5%<br><br>Diabetes mellitus (Age $\geq$70 years)<br>1.33 (0.78-2.28) I^2=0%<br><br>Diabetes mellitus (Age <70 years)<br>2.05 (1.44-2.94) I^2=32%
Wu, Zuo[76]<br><br>Inception to May 13, 2020<br><br>N=73 studies (171,108 patients)<br>54 China, 4 Italy, 2 USA, 2 UK, 2 France, 2 Spain, 6 'other'<br><br>Case-control/cohort<br><br>Agency for Healthcare Research and Quality, 31 high quality, 41 moderate, 1 low<br><br>AMSTAR2: Critically low quality	Hypertension<br>CVD<br>Arrhythmia<br>Renal disease<br>Smoker<br>Acute cardiac injury	Severe COVID	OR (95% CI)<br><br>Hypertension<br>N=22 studies<br>2.40 (2.08-2.78) I^2=39%<br><br>CVD<br>N=15 studies<br>3.54 (2.68-4.68) I^2=37%<br><br>Arrhythmia<br>N=3 studies<br>14.8 (8.9-24.6) I^2=49%<br><br>Renal disease<br>N=15 studies<br>1.84 (1.47-2.30) I^2=26%<br><br>Smoker<br>N=10 studies<br>1.61 (1.28-2.02) I^2=0%<br><br>Acute cardiac injury<br>N=7 studies<br>11.9 (7.64-18.46) I^2=0%<br><br>Incidence of acute cardiac injury<br>6% (3%-9%)
Xu, Mao[77]<br><br>Inception to March 8, 2020<br><br>N=20 studies (4,602 patients)<br>20 China<br><br>Cross-sectional<br>American Agency for Healthcare Research and Quality, range 3-7	BMI<br>Smoker<br>Diabetes mellitus<br>Hypertension<br>CVD	Severe COVID	SMD (95% CI)<br><br>BMI<br>3.38 (0.07-6.69)<br><br>OR (95% CI)<br><br>Smoker<br>N=3 studies (412 patients)<br>1.40 (0.65-3.01) I^2=0%<br><br>Diabetes mellitus<br>N=10 studies (1,083 patients)

AMSTAR2: Critically low quality			3.04 (2.01-4.60) I^2=20%<br><br>Hypertension<br>N=10 studies (1,083 patients)<br>2.31 (1.68-3.18) I^2=47%<br><br>CVD<br>N=7 studies (906 patients)<br>2.76 (1.39-5.45) I^2=26%
Youssef, Hussein[78]<br><br>Inception to April 16, 2020<br><br>N=20 studies (3,428 patients)<br>20 China<br><br>Case-control/cohort<br>Newcastle-Ottawa scale, 2-8 (1 study =2, 2 studies =3, 6 studies =5, 2 studies =6, 4 studies =7, 5 studies =8)<br><br>AMSTAR2: Moderate quality	Hypertension<br>Renal disease<br>Diabetes mellitus<br>CVD<br>Chronic liver disease<br>Cerebrovascular disease<br>Myocardial injury	Severe COVID	OR (95% CI)<br><br>Hypertension<br>2.36 (1.86-3.01) I^2=14%<br>N=13 studies (2,141 patients)<br><br>Renal disease<br>7.28 (3.25-16.16) I^2=0%<br>N=7 studies (1,675 patients)<br><br>Diabetes mellitus<br>2.72 (2.05-3.60) I^2=42%<br>N=14 studies (2,193 patients)<br><br>CVD<br>5.11 (2.03-12.83) I^2=77%<br>N=12 studies (2,327 patients)<br><br>Chronic liver disease<br>1.17 (0.66-2.06) I^2=0%<br>N=9 studies (1,629 patients)<br><br>Cerebrovascular disease<br>5.73 (2.52-13.04) I^2=33%<br>N=5 studies (769 patients)<br><br>Myocardial injury<br>11.2 (0.44-285.9) I^2=90%<br>N=3 studies (464 patients)
Yu, Wu[79]<br><br>December 2019 to July 25, 2020<br><br>N=31 studies (23,632 patients)<br>20 China, 4 USA, 2 Brazil, 1 Greece, 1 Iran, 1 Italy, 1 Spain, 1 Oman<br><br>Case-control/cohort<br>Newcastle-Ottawa scale, range 6-8 (5 studies =6, 19 studies =7, 7 studies =8)<br><br>AMSTAR2: Moderate quality	Cardio-cerebrovascular disease	Severe COVID<br>Mortality	OR (95% CI)<br><br>Cardio-cerebrovascular disease and severe COVID<br>N=20 studies<br>3.00 (2.10-4.30) I^2=65%<br><br>Cardio-cerebrovascular disease and mortality<br>N=16 studies<br>5.59 (2.81-11.11) I^2=94%

Zhang, Shen[80]<br><br>Inception to May 8, 2020<br><br>N=17 studies (1,913 patients)<br>6 China, 5 France, 3 Italy, 3 Netherlands<br><br>Case-control/cohort<br>Newcastle-Ottawa scale, range 5-8 (2 studies =5, 1 study =6, 8 studies =7, 6 studies =8)<br><br>AMSTAR2: Moderate quality	COVID-19<br><br><br><br><br><br><br><br>Venous thromboembolism	Incident:<br>- Venous thromboembolism<br>- Pulmonary embolism<br>- Deep vein thrombosis<br><br>Severe COVID	Incidence (95% CI)<br><br>Venous thromboembolism<br>0.25 (0.19-0.31) I^2=96%<br><br>Pulmonary embolism<br>0.19 (0.13-0.25) I^2=93%<br><br>Deep vein thrombosis<br>0.07 (0.04-0.10) I^2=88%<br><br>RR (95% CI)<br><br>Venous thromboembolism and severe COVID<br>4.76 (2.66-8.50) I^2=47%
Zhang, Wu[81]<br><br>Inception to March 20, 2020<br><br>N=12 studies (2,389 patients)<br>12 China<br><br>Case-control/cohort<br>Newcastle-Ottawa scale and STROBE, range 6-8 (6 studies =6, 7 studies =7, 5 studies =8)<br><br>AMSTAR2: Critically low quality	Hypertension	Severe COVID<br>Mortality	OR 95%CI<br><br>Hypertension and severe COVID<br>2.27 (1.80-2.86) I^2=8%<br><br>Stratified by age:<br><50 years<br>2.21 (1.58-3.10) I^2=0%<br>$\geq$50 years<br>2.32 (1.70-3.17) I^2=42%<br><br>Hypertension and mortality<br>3.48 (1.72-7.08) I^2=56%
Zhao, Meng[82]<br><br>December 2019 to March 22, 2020<br><br>N=11 studies (2,002 patients)<br>11 China<br><br>Case series<br>Methodological index non-randomized studies (MINORS) statement, rangre10-13 (overall quality moderate)<br><br>AMSTAR2: Critically low quality	Smoking history	Severe COVID	OR (95% CI)<br><br>Smoking history<br>1.98 (1.29-3.05) I^2=44%
Zheng, Peng[83]<br><br>January 1, 2020 to Mar 20, 2020<br><br>N=13 studies (3,027 patients)<br>13 China<br><br>Case-control/cohort	Current smoking<br>Diabetes mellitus<br>CVD<br>Hypertension	Composite outcome of severe COVID/mortality	OR (95% CI)<br><br>Current smoking<br>N=5 studies (1,980 patients)<br>2.04 (1.32-3.25) I^2=0%<br><br>Diabetes mellitus<br>N=11 studies (2,579 patients)

MINORS statement, range 18-21 (all low risk)<br><br>AMSTAR2: Low quality			3.68 (2.68-5.03) I^2=45%<br><br>CVD<br>N=10 studies (2,422 patients)<br>5.19 (3.25-8.29) I^2=37%<br><br>Hypertension<br>N=10 studies (2,527 patients)<br>2.72 (1.60-4.64) I^2=72%
Zuin, Rigatelli[84]<br><br>Inception to April 10, 2020<br><br>N=9 studies (1,686 patients)<br>Primary study origin NR<br><br>Primary study design NR<br>Newcastle-Ottawa Scale, 7 high quality<br><br>AMSTAR2: Low quality	COVID-19<br><br><br>Acute cardiac injury	Acute cardiac injury<br><br><br>Mortality	Incidence of acute cardiac injury<br>24%<br><br>OR (95% CI)<br><br>Acute cardiac injury and mortality<br>21.65 (8.60-54.52) I^2=82%
OR; Odds ratio, RR; Relative risk, SMD; Standardised mean difference, CI; Confidence interval, I^2; I-squared test for heterogeneity, AMSTAR2; a critical appraisal tool for systematic reviews, BMI; Body mass index, CVD; Cardiovascular disease, CAD; Coronary artery disease, CHD; Coronary heart disease, ICU; Intensive care unit, NR; not reported.<br>N=studies included in meta-analysis (n=patients)<br>Severe COVID-19 (as described in primary systematic reviews)			

Supplementary references

1. Aggarwal G, Cheruiyot I, Aggarwal S, et al. Association of Cardiovascular Disease With Coronavirus Disease 2019 (COVID-19) Severity: A Meta-Analysis. *Curr Probl Cardiol* 2020; 45: 100617.
2. Almeshari MA, Alobaidi NY, Asmri MA, et al. Mechanical ventilation utilization in COVID-19: A systematic review and meta-analysis. *medRxiv* 2020.
3. Alqahtani JS, Oyelade T, Aldhahir AM, et al. Prevalence, Severity and Mortality associated with COPD and Smoking in patients with COVID-19: A Rapid Systematic Review and Meta-Analysis. *PLOS ONE* 2020; 15: e0233147.
4. Bajgain KT, Badal S, Bajgain BB, et al. Prevalence of Comorbidities Among Individuals With COVID-19: A Rapid Review of current Literature. *Am J Infect Control* 2020.
5. Barrera FJ, Shekhar S, Wurth R, et al. Prevalence of Diabetes and Hypertension and Their Associated Risks for Poor Outcomes in Covid-19 Patients. *J Endocr Soc* 2020; 4: bvaa102.
6. Bennett S, Tafuro J, Mayer J, et al. Clinical features and outcomes of adults with COVID-19: A systematic review and pooled analysis of the literature. *Int J Clin Pract* 2020: e13725.
7. Bhatia R, Pedapati R, Komakula S, et al. Stroke in coronavirus disease 2019: A systematic review. *J Stroke* 2020; 22: 324-335.
8. Biswas M, Rahaman S, Biswas TK, et al. Effects of Sex, Age and Comorbidities on the Risk of Infection and Death Associated with COVID-19: A Meta-Analysis of 47807 Confirmed Cases. *SSRN* 2020.
9. Chang R, Elhusseiny K, Yeh Y, et al. COVID-19 ICU and Mechanical Ventilation Patient Characteristics and Outcomes - A Systematic Review and Meta-analysis. *ResearchSquare* 2020.
10. Chen Y, Gong X, Wang L, et al. Effects of hypertension, diabetes and coronary heart disease on COVID-19 diseases severity: a systematic review and meta-analysis. *medRxiv* 2020.
11. Chidambaram V, Tun NL, Haque WZ, et al. Predictors of Severity and Mortality Among Patients with Coronavirus Disease 2019: A Systematic Review and Meta-Analysis. *SSRN* 2020.
12. De Lorenzo A, Kasal DA, Tura BR, et al. Acute cardiac injury in patients with COVID-19. *Am J Cardiovasc Dis* 2020; 10: 28-33.
13. Fang X, Li S, Yu H, et al. Epidemiological, comorbidity factors with severity and prognosis of COVID-19: a systematic review and meta-analysis. *Aging* 2020; 12.
14. Figliozzi S, Masci PG, Ahmadi N, et al. Predictors of adverse prognosis in COVID-19: A systematic review and meta-analysis. *Eur J Clin Invest* 2020; 50: e13362.
15. Flook M, Jackson C, Vasileiou E, et al. Informing the public health response to COVID-19 (and lessons learnt for future pandemics): a systematic review of risk factors for disease, severity, and mortality. *ResearchSquare* 2020.
16. Florez-Perdomo WA, Serrato-Vargas SA, Bosque-Varela P, et al. Relationship between the history of cerebrovascular disease and mortality in COVID-19 patients: A systematic review and meta-analysis. *Clin Neurol Neurosurg* 2020; 197: 106183.
17. Fridman S, Bullrich MB, Jimenez-Ruiz A, et al. Stroke Risk, phenotypes, and death in COVID-19: Systematic review and newly reported cases. *Neurology* 2020.
18. Fu L, Wang B, Yuan T, et al. Clinical characteristics of coronavirus disease 2019 (COVID-19) in China: a systematic review and meta-analysis. *J Infect* 2020; 80: 656-665.
19. Gu ZC, Zhang C, Kong LC, et al. Incidence of myocardial injury in coronavirus disease 2019 (COVID-19): a pooled analysis of 7,679 patients from 53 studies. *Cardiovas Diagn Ther* 2020; 10: 667-677.
20. Gulsen A, Arpinar Yigitbas B, Uslu B, et al. The effect of smoking on COVID-19 symptom severity: Systematic review and meta-analysis. *medRxiv* 2020.
21. Hamam O, Goda A, Eldalal M, et al. Cardiac Arrhythmias in Patients with COVID-19: A Systematic review and Meta-analysis. *medRxiv* 2020.
22. Hammoud H, Bendari A, Bendari T, et al. Post mortem pathological findings in COVID-19 cases: A Systematic Review. *medRxiv* 2020.
23. Han P-l, Diao K-y, Pang T, et al. Comparison of Clinical Features between Critically and Non-Critically Ill Patients In SARS and COVID-19: A Systematic Review and Meta-Analysis. *SSRN* 2020.
24. Hessami A, Shamshirian A, Heydari K, et al. Cardiovascular diseases burden in COVID-19: Systematic review and meta-analysis. *Am J Emerg Med* 2020: S0735-6757(0720)30908-30906.
25. Hu Y, Sun J, Dai Z, et al. Prevalence and severity of corona virus disease 2019 (COVID-19): A systematic review and meta-analysis. *J Clin Virol* 2020; 127: 104371.
26. Islam MS, Barek MA, Aziz MA, et al. Association of age, sex, comorbidities, and clinical symptoms with the severity and mortality of COVID-19 cases: a meta-analysis with 85 studies and 67299 cases. *medRxiv* 2020.

27. Izcovich A, Ragusa MA, Tortosa F, et al. Prognostic Factors for Severity and Mortality in Patients Infected with COVID-19: A Systematic Review. *SSRN* 2020.
28. Jain V and Yuan JM. Predictive symptoms and comorbidities for severe COVID-19 and intensive care unit admission: a systematic review and meta-analysis. *Int J Public Health* 2020; 65: 533-546.
29. Khan MMA, Khan MN, Mustagir MG, et al. Effects of underlying morbidities on the occurrence of deaths in COVID-19 patients: A systematic review and meta-analysis. *J Glob Health* 2020; 10: 020503.
30. Kumar A, Aroray A, Sharma P, et al. Clinical Features of COVID-19 and Factors Associated with Severe Clinical Course: A Systematic Review and Meta-Analysis. *SSRN* 2020: 3566166.
31. Kumar A, Arora A, Sharma P, et al. Is diabetes mellitus associated with mortality and severity of COVID-19? A meta-analysis. *Diabetes Metab Syndr* 2020; 14: 535-545.
32. Li X, Guan B, Su T, et al. Impact of cardiovascular disease and cardiac injury on in-hospital mortality in patients with COVID-19: a systematic review and meta-analysis. *Heart* 2020; 106: 1142-1147.
33. Li J, He X, Yuanyuan N, et al. Meta-analysis investigating the relationship between clinical features, outcomes, and severity of severe acute respiratory syndrome coronavirus 2 (SARS-CoV-2) pneumonia. *Am J Infect Control* 2020.
34. Li J, Huang DQ, Zou B, et al. Epidemiology of COVID-19: A Systematic Review and Meta-analysis of Clinical Characteristics, Risk factors and Outcomes. *J Med Virol* 2020.
35. Liu H, Chen S, Liu M, et al. Comorbid Chronic Diseases are Strongly Correlated with Disease Severity among COVID-19 Patients: A Systematic Review and Meta-Analysis. *Aging Dis* 2020; 11: 668-678.
36. Liu Y-F, Zhang Z, Pan X-L, et al. The Chronic Kidney Disease and Acute Kidney Injury Involvement in COVID-19 Pandemic: A Systematic Review and Meta-analysis. *medRxiv* 2020.
37. Lu L, Zhong W, Bian Z, et al. A Comparison of Mortality-related Risk Factors of COVID-19, SARS, and MERS: A Systematic Review and Meta-analysis. *J Infect* 2020; 81: e18-e25.
38. Luo L, Fu M, Li Y, et al. The potential association between common comorbidities and severity and mortality of coronavirus disease 2019: A pooled analysis. *Clin Cardiol* 2020.
39. Ma C, Gu J, Hou P, et al. Incidence, clinical characteristics and prognostic factor of patients with COVID-19: a systematic review and meta-analysis. *medRxiv* 2020.
40. Mantovani A, Byrne CD, Zheng MH, et al. Diabetes as a risk factor for greater COVID-19 severity and in-hospital death: A meta-analysis of observational studies. *Nutr Metab Cardiovasc Dis* 2020; 30: 1236-1248.
41. Mao Y, Lin W, Wen J, et al. Clinical Characteristics, Prognosis and Death-related Factors of Diabetic Patients With COVID-19: A Systematic Review and Meta-analysis. *SSRN* 2020.
42. Matsushita K, Ding N, Kou M, et al. The relationship of COVID-19 severity with cardiovascular disease and its traditional risk factors: A systematic review and meta-analysis. *medRxiv* 2020.
43. Momtazmanesh S, Shobeiri P, Hanaei S, et al. Cardiovascular disease in COVID-19: a systematic review and meta-analysis of 10,898 patients and proposal of a triage risk stratification tool. *Egypt Heart J* 2020; 72: 41.
44. Moula AI, Micali LR, Matteucci F, et al. Quantification of Death Risk in Relation to Sex, Pre-Existing Cardiovascular Diseases and Risk Factors in COVID-19 Patients: Let's Take Stock and See Where We Are. *J Clin Med* 2020; 9: 1-17.
45. Nannoni S, de Groot R, Bell S, et al. EXPRESS: Stroke in COVID-19: a systematic review and meta-analysis. *Int J Stroke* 2020: 1747493020972922.
46. Nasiri MJ, Haddadi S, Tahvildari A, et al. COVID-19 clinical characteristics, and sex-specific risk of mortality: Systematic Review and Meta-analysis. *medRxiv* 2020.
47. Noor FM and Islam MM. Prevalence and Associated Risk Factors of Mortality Among COVID-19 Patients: A Meta-Analysis. *J Community Health* 2020.
48. Palaiodimos L, Chamorro-Pareja N, Karamanis D, et al. Diabetes is associated with increased risk for in-hospital mortality in patients with COVID-19: a systematic review and meta-analysis comprising 18,506 patients. *medRxiv* 2020.
49. Parohan M, Yaghoubi S, Seraji A, et al. Risk factors for mortality in patients with Coronavirus disease 2019 (COVID-19) infection: a systematic review and meta-analysis of observational studies. *Aging Male* 2020: 1-9.
50. Parveen R, Sehar N, Bajpai R, et al. Association of diabetes and hypertension with disease severity in covid-19 patients: A systematic literature review and exploratory meta-analysis. *Diabetes Res Clin Pract* 2020; 166: 108295-108295.
51. Patanavanich R and Glantz SA. Smoking is associated with worse outcomes of COVID-19 particularly among younger adults: A systematic review and meta-analysis. *medRxiv* 2020.
52. Patel U, Malik P, Shah D, et al. Pre-existing cerebrovascular disease and poor outcomes of COVID-19 hospitalized patients: a meta-analysis. *J Neurol* 2020.
53. Patel U, Malik P, Usman MS, et al. Age-Adjusted Risk Factors Associated with Mortality and Mechanical Ventilation Utilization Amongst COVID-19 Hospitalizations-a Systematic Review and Meta-Analysis. *SN Compr Clin Med* 2020; 2: 1740-1749.

54. Pôrto E, Iamonti V, Castro A, et al. Mortality in patients with diabetes by COVID 19 a systematic review. *ResearchSquare* 2020.
55. Reddy RK, Charles WN, Sklavounos A, et al. The effect of smoking on COVID-19 severity: a systematic review and meta-analysis. *J Med Virol* 2020.
56. Rhim H, Park J, Lee Y, et al. Clinical, laboratory, and radiologic findings associated with mortality in COVID-19: A systematic review and meta-analysis. *ResearchSquare* 2020.
57. Roncon L, Zuin M, Rigatelli G, et al. Diabetic patients with COVID-19 infection are at higher risk of ICU admission and poor short-term outcome. *J Clin Virol* 2020; 127: 104354.
58. Sabatino J, De Rosa S, Di Salvo G, et al. Impact of cardiovascular risk profile on COVID-19 outcome. A meta-analysis. *PLOS ONE* 2020; 15: e0237131.
59. Sales-Peres SHdC, de Azevedo-Silva LJ, Bonato RCS, et al. Coronavirus (SARS-CoV-2) and the risk of obesity for critically illness and ICU admitted: Meta-analysis of the epidemiological evidence. *Obes Res clin Pract* 2020; 14: 389-397.
60. Sepandi M, Taghdir M, Alimohamadi Y, et al. Factors Associated with Mortality in COVID-19 Patients: A Systematic Review and Meta-Analysis. *Iran J Public Health* 2020; 49: 1211-1221.
61. Shafi AMA, Shaikh SA, Shirke MM, et al. Cardiac manifestations in COVID-19 patients-A systematic review. *J Card Surg* 2020; 35: 1988-2008.
62. Shao M-J, Shang L-X, Luo J-Y, et al. Myocardial injury is associated with higher mortality in patients with coronavirus disease 2019: a meta-analysis. *J Geriatr Cardiol* 2020; 17: 224-228.
63. Shi C, Wang L, Ye J, et al. Predictors of mortality in patients with coronavirus disease 2019: a systematic review and meta-analysis. *ResearchSquare* 2020.
64. Sinclair JE, Zhu Y, Xu G, et al. The role of pre-existing chronic disease in cardiac complications from SARS-CoV-2 infection: A systematic review and meta-analysis. *medRxiv* 2020.
65. Sreenivasan J, Khan MS, Anker SD, et al. Cardiovascular Risk Factors and Complications in Patients Infected with COVID-19: A Systematic Review. *SSRN* 2020.
66. Ssentongo P, Ssentongo AE, Heilbrunn ES, et al. Association of cardiovascular disease and 10 other pre-existing comorbidities with COVID-19 mortality: A systematic review and meta-analysis. *PLOS ONE* 2020; 15: e0238215.
67. Tabrizi R, Lankarani KB, Nowrouzi-sohrabi P, et al. The role of comorbidities and clinical predictors of severe disease in COVID-19: a systematic review and meta-analysis. *medRxiv* 2020.
68. Tamara A and Tahapary DL. Obesity as a predictor for a poor prognosis of COVID-19: A systematic review. *Diabetes Metab Syndr* 2020; 14: 655-659.
69. Taylor EH, Hofmeyr R, Nejthardt M, et al. Risk factors and interventions associated with mortality or survival in adult covid-19 patients admitted to critical care: A systematic review and meta-analysis. *Southern African J Anaesth Analg* 2020; 26: 116-127.
70. Tian W, Jiang W, Yao J, et al. Predictors of mortality in hospitalized COVID-19 patients: A systematic review and meta-analysis. *J Med Virol* 2020; 92: 1875-1883.
71. Villalobos N, Ott J, Klett-Tammen C, et al. Quantification of the association between predisposing health conditions, demographic, and behavioural factors with hospitalisation, intensive care unit admission, and death from COVID-19: a systematic review and meta-analysis. *medRxiv* 2020.
72. Wang Z, Deng H, Ou C, et al. Clinical symptoms, comorbidities and complications features in severe and non-severe patients with COVID-19: a systematic review and meta-analysis without cases duplication. *ResearchSquare* 2020.
73. Wang B, Li R, Lu Z, et al. Does comorbidity increase the risk of patients with COVID-19: evidence from meta-analysis. *Aging* 2020; 12: 6049-6057.
74. Wu X, Liu L, Jiao J, et al. Characterization of clinical, laboratory and imaging factors related to mild vs. severe Covid-19 infection: a systematic review and meta-analysis. *Ann Med* 2020; 52: 1-21.
75. Wu ZH, Tang Y and Cheng Q. Diabetes increases the mortality of patients with COVID-19: a meta-analysis. *Acta Diabetol* 2020.
76. Wu T, Zuo Z, Kang S, et al. Multi-organ dysfunction in patients with COVID-19: A systematic review and meta-analysis. *Aging Dis* 2020; 11: 874-894.
77. Xu L, Mao Y and Chen G. Risk factors for severe corona virus disease 2019 (COVID-19) patients : a systematic review and meta analysis. *medRxiv* 2020.
78. Youssef M, H Hussein M, Attia AS, et al. COVID-19 and liver dysfunction: A systematic review and meta-analysis of retrospective studies. *J Med Virol* 2020; 92: 1825-1833.
79. Yu JN, Wu BB, Yang J, et al. Cardio-Cerebrovascular Disease is Associated With Severity and Mortality of COVID-19: A Systematic Review and Meta-Analysis. *Biol Res Nurs* 2020.
80. Zhang C, Shen L, Le KJ, et al. Incidence of Venous Thromboembolism in Hospitalized Coronavirus Disease 2019 Patients: A Systematic Review and Meta-Analysis. *Front Cardiovasc Med* 2020; 7: 151.

81. Zhang J, Wu J, Sun X, et al. Association of hypertension with the severity and fatality of SARS-CoV-2 infection: A meta-analysis. *Epidemiol Infect* 2020; 148: e106.
82. Zhao Q, Meng M, Kumar R, et al. The impact of COPD and smoking history on the severity of Covid-19: A systemic review and meta-analysis. *J Med Virol* 2020.
83. Zheng Z, Peng F, Xu B, et al. Risk factors of critical & mortal COVID-19 cases: A systematic literature review and meta-analysis. *J Infect* 2020; 81: e16-e25.
84. Zuin M, Rigatelli G, Zuliani G, et al. Incidence and mortality risk in coronavirus disease 2019 patients complicated by acute cardiac injury: systematic review and meta-analysis. *J Cardiovasc Med* 2020; 21: 759-764.
